# Hydrogen sulfide promotes flowering in heading Chinese cabbage by S-sulfhydration of BraFLCs

**DOI:** 10.1038/s41438-020-00453-3

**Published:** 2021-02-01

**Authors:** Xiaoli Ma, Liping Zhang, Zhuoya Pei, Linlin Zhang, Zhiqiang Liu, Danmei Liu, Xuefeng Hao, Zhuping Jin, Yanxi Pei

**Affiliations:** 1 School of Life Science and Shanxi Key Laboratory for Research and Development of Regional Plants, Shanxi University, Taiyuan, Shanxi Province 030006 China; 2 Department of Biological Science and Technology, Jinzhong University, Jinzhong, Shanxi Province 030619 China; 3 Department of Biology, Taiyuan Normal University, Jinzhong, Shanxi Province 030619 China

**Keywords:** Non-model organisms, Reproductive biology, Transcriptional regulatory elements

## Abstract

Heading Chinese cabbage (*Brassica rapa* L. syn. *B*. *campestris* L. ssp. *chinensis* Makino var. *pekinensis* (Rupr.) J. Cao et Sh. Cao) is a cruciferous *Brassica* vegetable that has a triplicate genome, owing to an ancient genome duplication event. It is unclear whether the duplicated homologs have conserved or diversified functions. Hydrogen sulfide (H_2_S) is a plant gasotransmitter that plays important physiological roles in growth, development, and responses to environmental stresses. The modification of cysteines through S-sulfhydration is an important mechanism of H_2_S, which regulates protein functions. H_2_S promotes flowering in *Arabidopsis* and heading Chinese cabbage. Here we investigated the molecular mechanisms of H_2_S used to promote flowering in the latter. Four, five, and four *BraFLC*, *BraSOC I*, and *BraFT* homologs were identified in heading Chinese cabbage. Different BraFLC proteins were bound to different CArG boxes in the promoter regions of the *BraSOC I* and *BraFT* homologs, producing different binding patterns. Thus, there may be functionally diverse *BraFLC* homologs in heading Chinese cabbage. Exogenous H_2_S at 100 μmol L^−1^ significantly promoted flowering by compensating for insufficient vernalization. BraFLC 1 and BraFLC 3 underwent S-sulfhydration by H_2_S, after which their abilities to bind most *BraSOC I* or *BraFT* promoter probes weakened or even disappeared. These changes in binding ability were consistent with the expression pattern of the *BraFT* and *BraSOC I* homologs in seedlings treated with H_2_S. These results indicated that H_2_S signaling regulates flowering time. In summary, H_2_S signaling promoted plant flowering by weakening or eliminating the binding abilities of BraFLCs to downstream promoters through S-sulfhydration.

## Introduction

Heading Chinese cabbage (*Brassica rapa* L. syn. *B*. *campestris* L. ssp. *chinensis* Makino var. *pekinensis* (Rupr.) J. Cao et Sh. Cao) is an important vegetable crop in East Asia. *Arabidopsis* and heading Chinese cabbage diverged from a common ancestor and evolved into unique species. Comparative genomics research has provided a comprehensive and in-depth understanding of the genomic structural characteristics of *Brassica* crops, and there is obvious collinearity between the heading Chinese cabbage and *Arabidopsis* genomes^1^. During evolution, the heading Chinese cabbage genome became a triploid genome through genome duplication^2^. Consequently, there are often multiple homologs of *Arabidopsis* genes in Chinese cabbage. However, this collinear relationship is not a perfect 3 : 1 ratio^3^. Currently, more gene functional research is undertaken using heading Chinese cabbage than *Arabidopsis*. One goal is to determine whether the multiple homologs in heading Chinese cabbage have functional redundancy and whether their functions are similar to those of their *Arabidopsis* counterparts.

Flowering is a key developmental event in plants and is part of the transition from vegetative to reproductive growth. The responses of the flowering process to light, temperature, and other environmental conditions have been used to divide the flowering regulatory pathways into the photoperiodic, autonomous, vernalization, and gibberellin (GA) pathways in *Arabidopsis*^4,5^. Various regulatory pathways involve the integration factor genes *Suppressor of Overexpression of Constant 1* (*SOC I*), *Flowering Locus T* (*FT*) and *LEAFY*, which regulate flowering time in *Arabidopsis*. Flowering Locus C (FLC), a MADS box transcription factor, integrates the autonomous and vernalization pathways by controlling *SOC I*, and *FT* has a circuit-resistance role across the whole flowering control network^6^. FLC also strongly inhibits the expression of genes that promote flowering in plants, such as *FT* and *SOC I*^2,7,8^. SOC I is also a MADS box transcription factor. This molecule integrates various flowering signals that are dependent on photoperiod, temperature, and hormones, and it is highly conserved in monocotyledons and dicotyledons. SOC I is a strong promoter of flowering and is downstream of many pathways that regulate flowering^9,10^. FT is at the core of a pathway that promotes flowering in response to changes in day length and triggers flowering when it is transmitted from the leaves to the shoot apex. It is considered to be the florigen or at least an important component of florigen^11^.

Vernalization is an essential process in some plants, especially winter plants, because it promotes flowering after a period of continuous low temperature. The *FLC* gene plays a pivotal role in vernalization^12^. The *FLC* gene in *Arabidopsis* is highly expressed until there is a period of continuous low temperature. This process is related to a series of active histone markers, such as H3K4me3 and H3K36me3^13^. In addition, some histone markers, such as histone deacetylation, H3K9me2, H3K27me3, and H4R3sme2, inhibit *FLC* expression^14–16^. However, there have been no reports that modifications of the FLC protein itself affect its function.

Over the past two decades, the unique and irreplaceable role of gasotransmitters in animals has been gradually elucidated. Hydrogen sulfide (H_2_S) was the third gasotransmitter recognized after nitric oxide and carbon monoxide^17^. Research on the role of H_2_S in plants has also progressed over the past decade. To date, research on the physiological functions of endogenous H_2_S has mainly focused on responses to abiotic stresses and their effects on growth and development. H_2_S enhances the tolerance of plants to drought, heavy metals, cold and heat, and other stresses. This molecule also promotes seed germination and adventitious root formation, improves plant photosynthetic efficiency, and inhibits petiole abscission^17–20^. However, to date, there have been no reports on the regulatory effects of H_2_S on flowering time in plants.

Uncovering the mechanisms underlying H_2_S functions is an important research area. Currently, the covalent modification of proteins by H_2_S is believed to be the most important process. This molecule changes the function of a protein through the S-sulfhydration (or persulfidation) of cysteine (Cys) residues^18,21,22^. At least 5% of *Arabidopsis* proteins may be S-sulfhydrated by physiological concentrations of H_2_S. These proteins are involved in many important processes, such as carbon metabolism, abiotic and biological stress responses, growth and development, and RNA translation^22^. However, there is currently little in-depth and systematic experimental evidence for this hypothesis.

Previously, we observed that H_2_S significantly promoted *Arabidopsis* flowering. In this study, we explored whether H_2_S has the same physiological functions in the flowering process of heading Chinese cabbage and the molecular mechanisms underlying its functions were also investigated.

## Results

### Exogenous physiological H2S promoted flowering

**Fig. 1:**
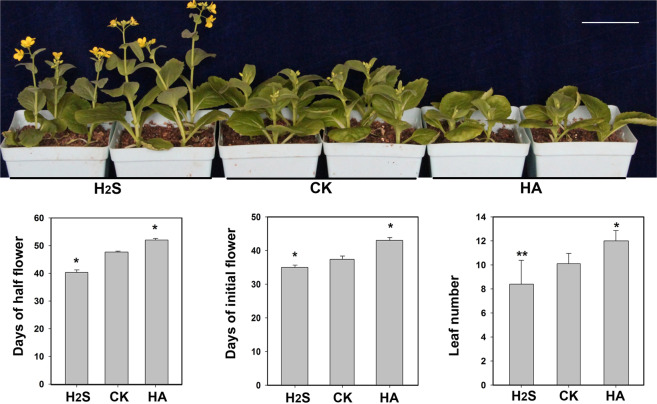
Effect of H2S on heading Chinese cabbage flowering. Phenotypes of plants treated with 100 μmol L^−1^ H_2_S or 2 mmol L^−1^ HA; *p < 0.05, **p < 0.01, Student’s *t*-test; bar = 4 cm

In previous studies, we observed that H_2_S promoted flowering in *Arabidopsis*. To explore whether H_2_S has the same effect in heading Chinese cabbage, we sprayed planted seedlings with different physiological concentrations of H_2_S (from 0 to 100 μmol L^−1^) until flowering. The results showed that 100 μmol L^−1^ significantly promoted flowering in heading Chinese cabbage (Suppl. Fig. 1). Therefore, we used 100 μmol L^−1^ in the subsequent experiments. We further confirmed that the early onset of flowering in heading Chinese cabbage under H_2_S treatment was indeed caused by the application of exogenous H_2_S by verifying the results using hydroxylamine (HA), which is an inhibitor of H_2_S synthesis in cells^23^. The results showed that the days to initial flower appearance, the days to half flowering, and the bolting leaf number for plants in the H_2_S treatment group were significantly lower than those in the control check (CK) group (Fig. 1). However, the flowering time of the HA treatment group was significantly delayed compared to that of the CK group. These results indicated that exogenous application of 100 μmol L^−1^ H_2_S can promote early flowering in heading Chinese cabbage.

### H_2_S rescued late flowering caused by insufficient vernalization

**Fig. 2:**
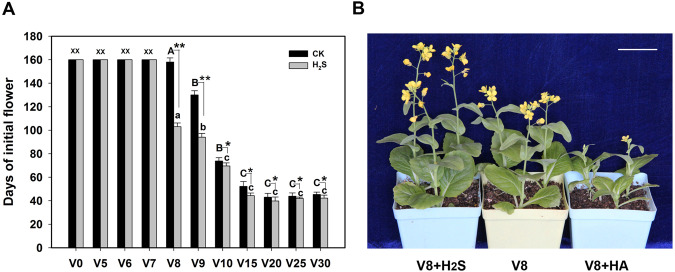
Effect of H2S on plant flowering time with insufficient vernalization. **A** Effects of different vernalization days on the appearance of initial flowers in heading Chinese cabbage with or without 100 µmol L^−1^ H_2_S treatment. **B** Effect of H_2_S on plant flowering under insufficient vernalization; V8 + H_2_S: insufficient vernalization + H_2_S treatment; V8: insufficient vernalization; V8 + HA: insufficient vernalization + HA treatment. V0, V5, V6, V7, V8, V9, V10, V15, V20, V25, and V30 indicate the different vernalization times (days); asterisks and letters above the bars indicate significant differences compared to CK (*p < 0.05; **p < 0.01; Student’s *t*-test); × indicates flowering was not observed until the 160th day; bar = 4 cm

Winter plants can flower earlier if they have undergone a suitable vernalization period. Therefore, we analyzed the effect of different vernalization times combined with H_2_S treatment to investigate whether the H_2_S-mediated promotion of flowering time in heading Chinese cabbage was related to the vernalization pathway. Figure 2A shows that when the vernalization treatment was performed at 4 °C for 0–7 days, the plants did not bloom regardless of whether H_2_S was applied (observed for 160 days). When the vernalization treatment lasted for more than 8 days, the flowering time gradually became earlier as the low-temperature period increased. The flowering time of the H_2_S-treated plants was significantly earlier than that of the CK plants (without H_2_S applied). Furthermore, when the vernalization treatment lasted 8–9 days, the effect of H_2_S was extremely significant (Fig. 2A, B). These results indicated that exogenous H_2_S applications at physiological concentrations could promote flowering by compensating for insufficient vernalization to some extent.

### Effect of H_2_S on the photoperiod pathway of flowering

**Fig. 3:**
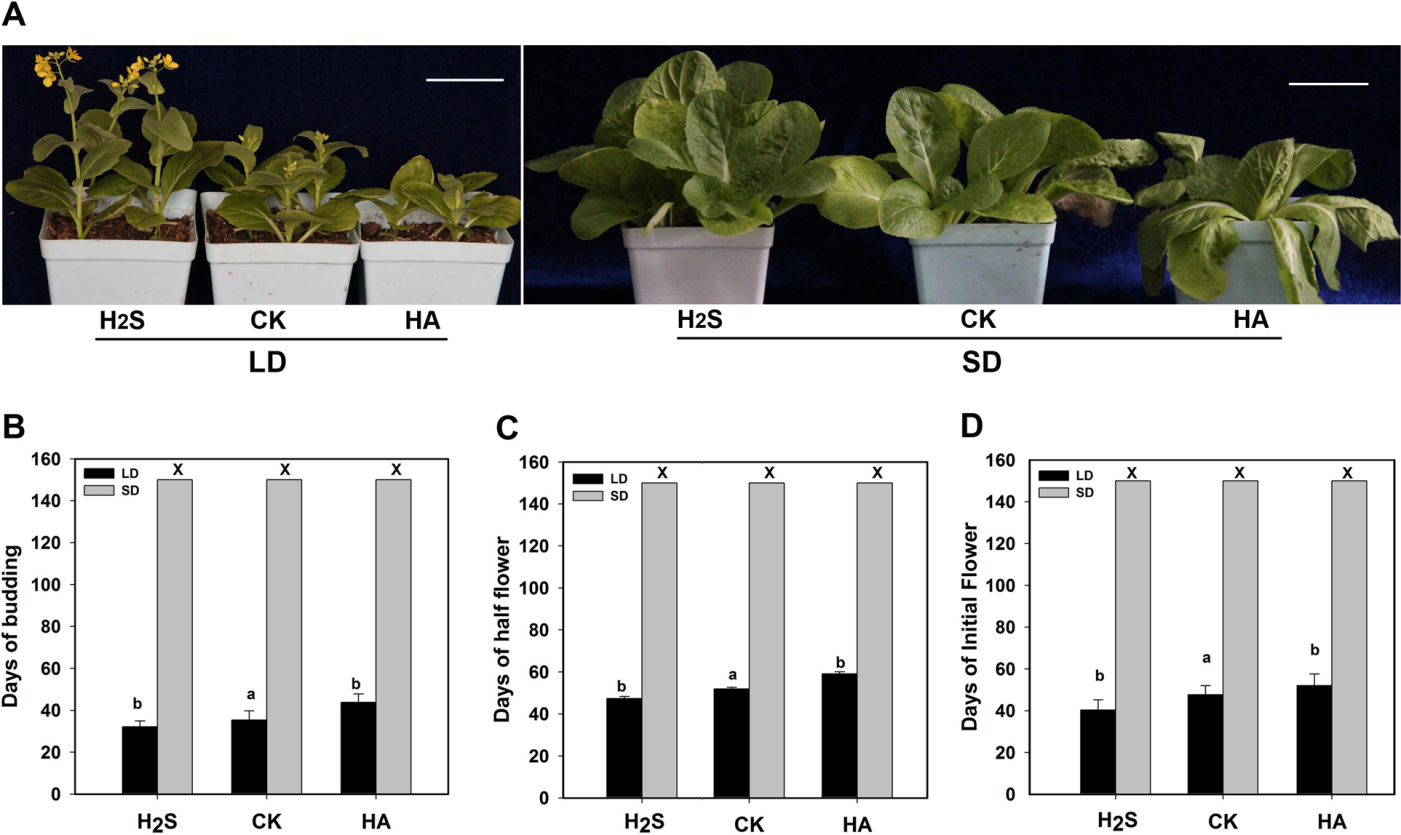
Effects of H2S on plant flowering under LD and SD conditions. **A** Phenotypes of the plants treated with 100 µmol L^−1^ H_2_S or 2 mmol L^−1^ HA under LD or SD conditions. **B**–**D** Days to budding, days to initial flower appearance, and days to half flowering in heading Chinese cabbage under LD and SD conditions. Letters above the bars indicate significant differences compared to CK (*p* < 0.05, Student’s *t*-test); × indicates flowering was not observed; bars = 4 cm

The photoperiod pathway, also known as the long-day pathway, is one of the most important flowering pathways. We carried out H_2_S and HA treatments under long and short daylight conditions (LD and SD, respectively) to observe the flowering changes in heading Chinese cabbage, to determine whether H_2_S was involved in the photoperiod pathway of flowering time. The results showed that H_2_S promoted flowering and that HA inhibited flowering under LD conditions (Fig. 3A left column). However, under SD conditions, the plant flowering time in the H_2_S, HA, and CK groups was severely delayed and no flowering was observed until the 160th day. In terms of the above results, it was still unclear whether H_2_S was involved in the photoperiod pathway. In addition, under SD conditions, the plants treated with H_2_S grew more vigorously than the CK plants. The HA treatment group confirmed this result (Fig. 3A right column).

### Effect of H_2_S on the GA pathway-mediated promotion of flowering

**Fig. 4:**
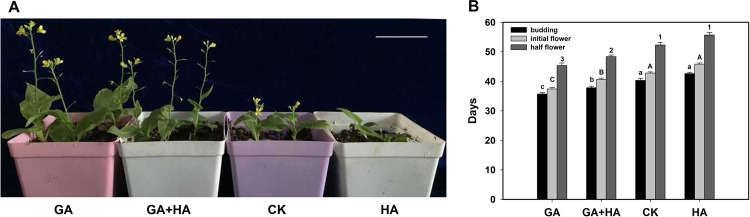
Effects of HA and GA3 on flowering of heading Chinese cabbage. **A** Phenotypes of heading Chinese cabbage treated with 300 mg L^−1^ GA_3_, 300 mg L^−1^ GA + 2 mmol L^−1^ HA, and 2 mmol L^−1^ HA for 10 days after planting. **B** Days to budding, initial flower appearance, and half to flowering in the GA_3_, GA_3_ + HA, and HA treatment groups compared to the CK groups. Numbers and letters above the bars indicate significant differences compared to CK (*p* < 0.05, Student’s *t*-test). Bar = 4 cm

GA is an important phytohormone that promotes plant flowering. Therefore, we analyzed the effects of GA and HA on flowering time to detect whether the flowering promotion effect of H_2_S was mediated by the GA pathway. The results showed that the GA_3_-treated plants flowered significantly earlier than the CK plants. Furthermore, GA_3_ could still promote plant flowering in the GA_3_ plus HA treatment group (Fig. 4). This result indicated that the absence of H_2_S did not affect the promotion of flowering by GA_3_. Therefore, H_2_S is not involved in the GA pathway.

### Cloning, expression, and purification of *BraFLC* homologous genes

The above data showed that H_2_S regulated heading Chinese cabbage flowering time via the vernalization pathway, although it was unclear whether it was the only way. In *Arabidopsis*, AtFLC is a key factor in the vernalization process. We therefore tried to reveal the relationship between H_2_S and *BraFLCs*, although it was very difficult due to the duplicate homologs in heading Chinese cabbage compared with *Arabidopsis*. The number of *BraFLC* genes in heading Chinese cabbage and whether they were functionally conserved or diversified were unclear. The coding domain sequences of the four *BraFLC* homologs were obtained from the Brassica Database (http://brassicadb.org/brad/) and named *BraFLC 1*, *BraFLC 2*, *BraFLC 3*, and *BraFLC 4*. After multiple alignment, the similarity of the four sequences was more than 83.91% and they contained open reading frames that were 621, 591, 594, and 432 bp in length (Suppl. Fig. 2). Further analysis of the BraFLC proteins using the SMART tool (http://smart.embl-heidelberg.de/) and a comparison with the AtFLC protein demonstrated that BraFLC 1, BraFLC 2, BraFLC 3, and AtFLC contained a MADS box and a K-box located at the N terminus, whereas the K-box was missing in BraFLC 4 (Suppl. Fig. 3A). A phylogenetic analysis using DNAMAN is shown in Suppl. Fig. 3B. The function of *BraFLCs* was further analyzed by constructing and identifying the prokaryotic expression vector pET28b-BraFLCs (Suppl. Fig. 4A). BL21 (DE3), containing pET28b-BraFLCs, was induced by 1 mmol L^−1^ isopropyl β-d-1-thiogalactopyranoside and incubated at 20 °C for 16 h. The target proteins were purified by nickel-chelating resin (Suppl. Fig. 4B).

### Analysis of the binding patterns for BraFLCs with the *BraSOC I* and *BraFT* promoters

*AtSOC I* and *AtFT* are all important downstream genes regulated by AtFLC. We obtained the promoter sequence for the five *BraSOC I* and four *BraFT* homologs (1500 and 2000 bp sequences upstream of the translation start site, respectively) from the Brassica Database (Suppl. Table 1). PlantCARE analysis (http://bioinformatics.psb.ugent.be/webtools/plantcare/html/) showed that the five promoters of the *BraSOC I* genes and the four promoters of the *BraFT* genes included both conservative domains and unique domains (Suppl. Fig. 5). The PlantCARE and PLACE databases (https://sogo.dna.affrc.go.jp/cgi-bin/sogo.cgi?lang=en & pj=640 & action=page & page=newplace) were used to analyze the homologous promoter sequences. A total of eight and six CArG boxes (FLC-binding sites) were found in the *BraSOC I* and *BraFT* promoters, respectively. These sequences are shown in Suppl. Fig. 6. All the CArG box-binding site sequences were cloned via specific primer PCR amplification (Suppl. Fig. 7) and acted as probe templates for the subsequent DNA–protein binding assay.

**Fig. 5:**
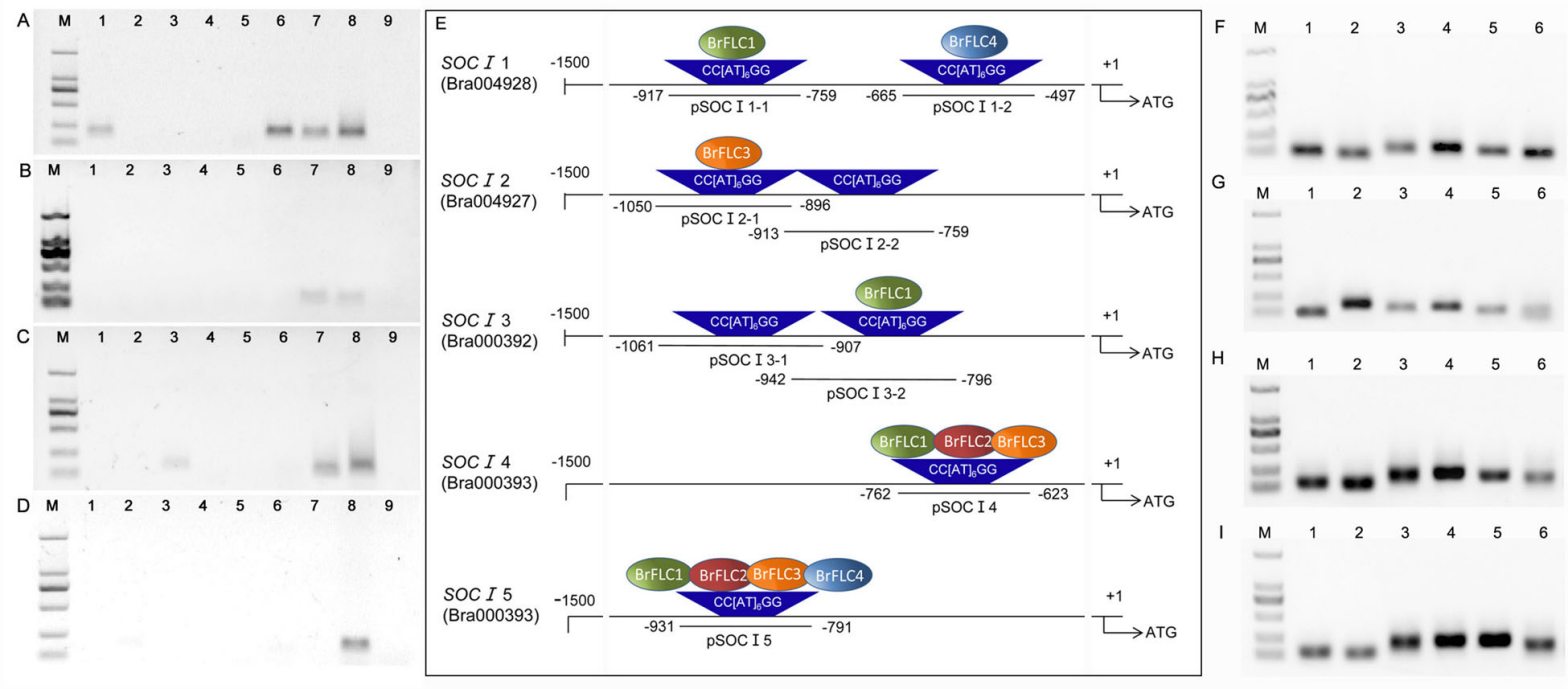
Binding patterns of BraFLCs with promoter probes of BraSOC I and BraFT homologs. **A**–**D** Binding reactions of BraFLC 1, BraFLC 2, BraFLC 3, and BraFLC 4 with eight *BraSOC I* promoter probes; M, molecular weight marker; Lanes 1–8: eight probes of *BraSOC I* promoters, pSOC I 1-1, pSOC I 1-2, pSOC I 2-1, pSOC I 2-2, pSOC I 3-1, pSOC I 3-2, pSOC I 4, and pSOC I 5, binding with BraFLCs; Lane 9: negative control. **E** Schematic illustration of the interactions between BraFLCs and pSOC I probes. The different colored balls represent the four different BraFLCs; the trapezoids indicate the CArG box locus of the five different *BraSOC I* promoter regions; the short lines indicate the DNA probe sites in the promoter region of the *BraSOC I* homologs. **F**–**I** Binding reactions of BraFLC 1, BraFLC 2, BraFLC 3, and BraFLC 4 with six *BraFT* promoter probes, respectively; M, molecular weight marker; Lanes 1–6: Six probes of *BraFTs*, pFT 1, pFT 2, pFT 3-1, pFT 3-2, pFT 4-1, and pFT 4-2, binding with BraFLCs

To determine the differentiation among the four *BraFLC* duplicated genes, we analyzed their binding patterns with the promoters of the five downstream *BraSOC I* homologs (Fig. 5A–D). The results clearly showed that the four BraFLC proteins had different patterns of binding with the eight CArG boxes in the *BraSOC I* promoters. The binding mode is summarized as a schematic illustration in Fig. 5E. For the promoters of *BraFTs*, all the BraFLC proteins bound all six CArG box probes (Fig. 5F–I). These results suggested functional differentiation in the BraFLCs.

### S-sulfhydration and functional regulation of BraFLCs mediated by H_2_S

**Fig. 6:**
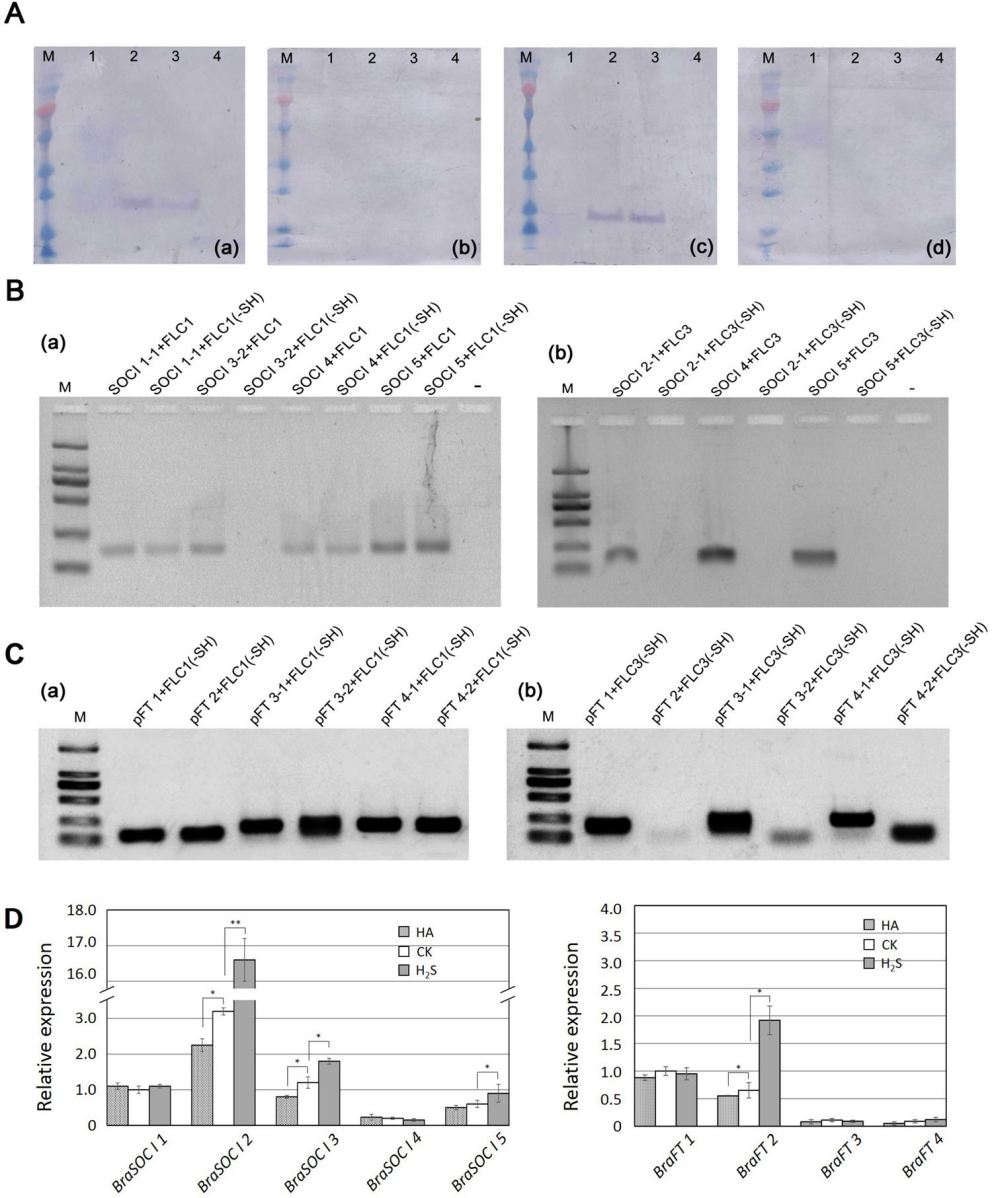
Effect of S-sulfhydration on BraFLC function. **A** S-sulfhydration modification of BraFLCs mediated by H_2_S. a S-sulfhydration of BraFLC 1 by H_2_S; b S-sulfhydration of BraFLC 2 by H_2_S; c S-sulfhydration of BraFLC 3 by H_2_S; d S-sulfhydration modification of BraFLC 4 by H_2_S; M: marker; 1, control, FLC + ddH_2_O; 2, BraFLC treated with 200 µmol L^−1^ NaHS; 3, BraFLC treated with 100 µmol L^−1^ NaHS; 4: negative control, DTT (protein + 1 mM DTT). Experimental procedure: the protein was treated with H_2_S, followed by MMTS, methyl methanethiosulfonate blocking, biotin-HPDP, N-(6-(biotinamido)hexyl)-3′-(2′-pyridyldithio)-propionamide biotin labeling, SDS-PAGE electrophoresis, and transmembrane treatment. Then, the samples were blocked with BSA, subjected to an anti-biotin primary antibody, an AP goat anti-mouse alkaline phosphatase-labeled sheep secondary antibody, and a NBT/BCIP, nitro blue tetrazolium/5-bromo-4-chloro-3-indolyl Phosphate color reaction assay. **B** Effects of S-sulfhydration on the binding ability of BraFLCs with *BraSOC I* promoter probes. a The binding ability of S-sulfhydrated BraFLC 1 and b the binding ability of S-sulfhydrated BraFLC 3; M: marker; SOC I 1-1 + FLC1: assay for SOC I 1-1 probes binding with BraFLC 1 without H_2_S treatment, and SOC I 1-1 + FLC1 (-SH): assay for SOCI 1-1 probe binding with S-sulfhydrated BraFLC 1; the representative symbol for other *BraSOC I* probes binding with FLC protein are the same as above; “−” negative control. **C** Effects of S-sulfhydration on the binding ability of BraFLCs with *BraFT* promoter probes. a The binding ability of S-sulfhydrated BraFLC 1 and b the binding ability of S-sulfhydrated BraFLC 3; M: marker; pFT 1 + FLC1: assay for pFT 1 probe binding with BraFLC 1 without H_2_S treatment, and pFT 1 + FLC1 (-SH): assay for pFT 1 probe binding with S-sulfhydrated BraFLC 1. The representative symbol for the other *BraFT* probes binding with BraFLC protein are the same as above; “−” negative control. **D** *BraSOC I* and *BraFT* homolog expression levels revealed by qRT-PCR analysis after treatment with 100 µmol L^−1^ NaHS solution or 2 mmol L^−1^ HA. Values are the mean ± SE of three biological replicates; *p < 0.05, **p < 0.01, Student’s *t*-test

The four BraFLCs-His tag proteins were purified from *Escherichia coli* BL21(DE3). The in vitro S-sulfhydration reaction was carried out using H_2_S as the sulfhydryl agent followed by detection using the biotin-switch method, as described previously^24^. The results showed that S-sulfhydration occurred in BraFLC 1 and BraFLC 3 after treatment with H_2_S. The western blotting signal clearly showed that the S-sulfhydration degree increased as the H_2_S concentration rose (Fig. 6A(a), (c)). However, no S-sulfhydration was detected in BraFLC 2 and BraFLC 4, as shown in Fig. 6A(b), (d). These data indicated that H_2_S could selectively cause S-sulfhydration in BraFLC members. We compared the binding ability of S-sulfhydrated BraFLC 1 and BraFLC 3 with the eight CArG box probes in *BraSOC I* homologs to determine whether S-sulfhydration could affect the binding abilities of transfactors with downstream promoters. The results showed that S-sulfhydration significantly changed the binding abilities of the BraFLC 1/3 proteins with the CArG box probes (Fig. 6B, C). After S-sulfhydration modification, the binding of BraFLC 1 to the pSOC I 1-1 probe was weakened and pSOC I 3-2 was unable to bind at all. However, this modification had no obvious effect on the ability of BraFLC 1 to bind with the pSOC I 4 or pSOC I 5 probes, as shown in Fig. 6B(a). S-sulfhydrated BraFLC 3 was unable to bind with the pSOC I 2, pSOC I 4, and pSOC I 5 probes (Fig. 6B(b)). The binding ability of S-sulfhydrylated BraFLC 3 with the pFT 2, pFT 3-2, and pFT 4-2 probes was abolished (Fig. 6C(b)), whereas S-sulfhydration had no influence on the binding ability of BraFLC 1 (Fig. 6C(a)).

To further confirm the effect of the covalent modification on the function of BraFLCs, we analyzed the expression patterns of the *BraFT* and *BraSOC I* homologs in seedlings treated with H_2_S and HA. The results showed that the expression levels of *BraSOC I* 2/3/5 and *BraFT 2* were upregulated, especially that of *BraSOC I* 2, whereas HA had the opposite effect. This expression pattern is basically consistent with the binding pattern of S-sulfhydrylated BraFLCs with downstream promoters. The above results clearly revealed that S-sulfhydration of BraFLCs caused by H_2_S can change their binding ability with some downstream promoters. This mechanism may be an important way that H_2_S affects the function of these transcription factors.

## Discussion

### Role of H_2_S in the flowering pathway of heading Chinese cabbage

Biennial plants and most winter annual plants need to undergo a long period of low-temperature vernalization before flowering. We observed the effects of H_2_S on flowering in heading Chinese cabbage (Fig. 1). Determining whether the promotional effects of H_2_S on heading Chinese cabbage flowering are related to vernalization is an important area of research. H_2_S promotes plant flowering, especially when low-temperature vernalization is not sufficient (Fig. 2). This finding strongly suggested that exogenous H_2_S cannot replace vernalization entirely; however, it can remedy insufficient vernalization, to a certain extent, in promoting flowering. In *Arabidopsis*, mutants of genes belonging to the photoperiod flowering pathway show the late-flowering phenotype under long-day (LD) conditions, whereas their phenotypes are similar to those of the wild type under short-day (SD) conditions^25^. The experimental data clearly showed that exogenous H_2_S promoted flowering in heading Chinese cabbage under LD conditions but not under SD conditions (Fig. 3). This result implied that H_2_S was involved in the regulation of flowering through the photoperiod pathway. In addition, during the cultivation period, plants in the H_2_S-treated group showed vigorous growth under SD conditions, whereas plants in the HA-treated group showed poor growth. This finding may be because H_2_S promotes photosynthesis^26^. The experimental data also showed that the absence of H_2_S did not affect the ability of GA_3_ to promote flowering (Fig. 4). Therefore, H_2_S is not a necessary downstream part of the GA pathway. However, it is not clear whether H_2_S affects the synthesis of endogenous GA from its upstream position and this issue should be investigated further. There is also an autonomous flowering pathway in *Arabidopsis* that is independent of the vernalization and photoperiod pathways^25^. The heading Chinese cabbage plants in this study did not flower under SD conditions, which made it difficult to determine whether H_2_S regulated flowering through the autonomous pathway. Such an investigation will require further analyses using heading Chinese cabbage varieties that flower earlier under SD conditions than those used in this study.

### Molecular mechanisms underlying the effects of H_2_S on flowering time

**Fig. 7:**
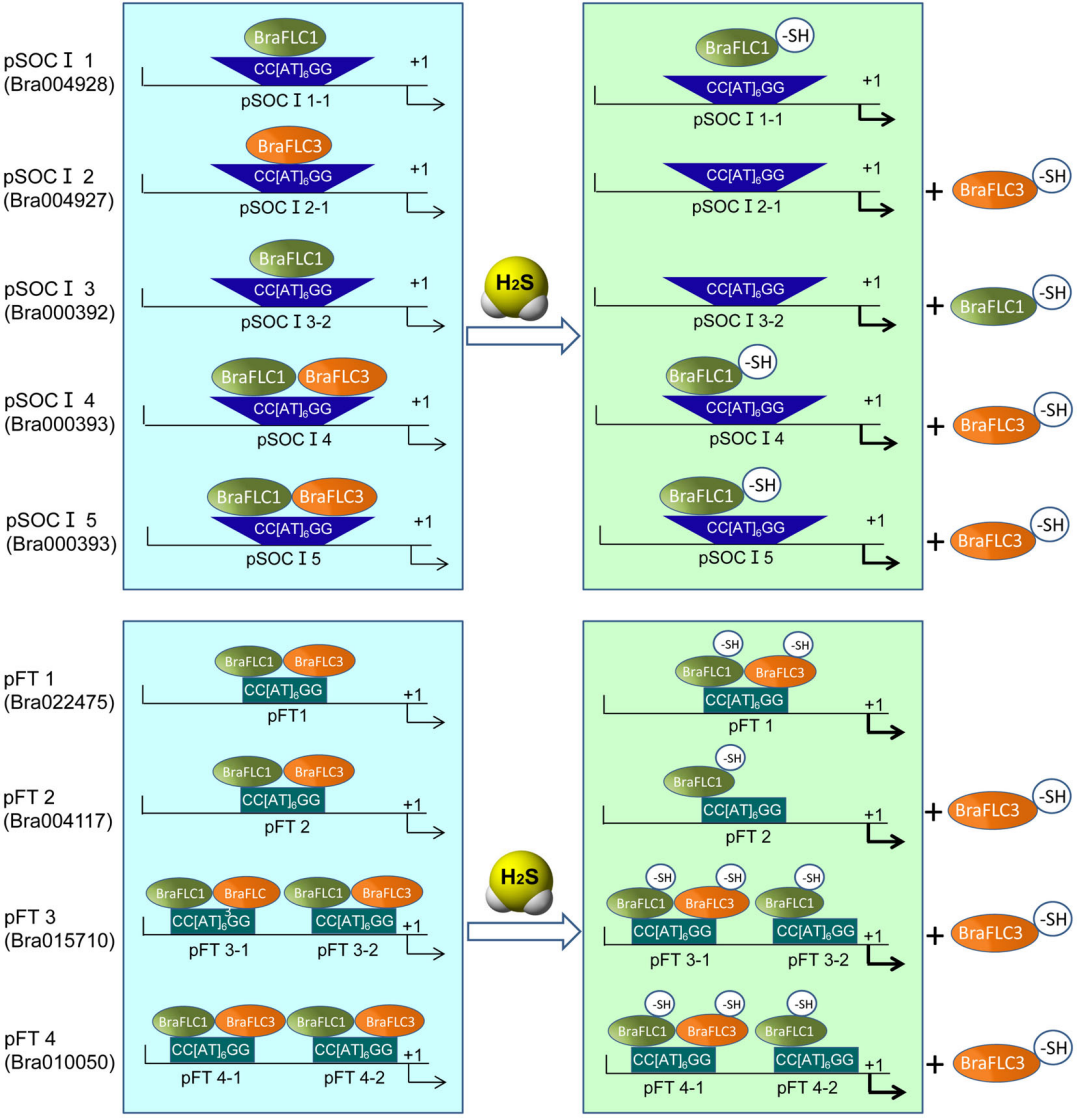
A model of H_2_S-promoted flowering *via* BraFLCs. H_2_S can promote flowering by regulating the binding abilities of BraFLCs to the downstream promoters through S-sulfhydration modification. The different colored balls indicate the different BraFLCs; the blue trapezoids and green rectangles represent the CArG boxes in the different *BraSOC I* (up panel) or *BraFT* (down panel) promoter regions, respectively

Many reports have confirmed the physiological functions of H_2_S, but its working mechanisms are still unclear. H_2_S modifies the Cys residues in proteins in cells, which is called S-sulfhydration (or persulfation). A sulfhydryl group (R-SH) is converted to a persulfide group (R-SSH) in Cys, which leads to an increase in Cys activity^27^. In *Arabidopsis*, this modification can cause structural, activity, or subcellular localization changes to target proteins, such as ascorbate peroxidase, glyceraldehyde-3-phosphate dehydrogenase, and glutamine synthetase^21,22^. Our previous data showed that low-temperature, high-temperature, salt, osmotic, and UV stresses and the H_2_S physiological concentration increased the overall S-sulfhydration levels in *Setaria italica* L.^28^. In this study, H_2_S selectively S-sulfhydrated BraFLC members, which led to significant changes in the BraFLC 1- and BraFLC 3-binding abilities (Fig. 6B, C). This result strongly implied that H_2_S regulated flowering time through BraFLCs. Histone methylation may activate or inhibit *AtFLC* expression^13,15^. To date, there have been no reports on any FLC functional changes owing to the modification of the protein itself. The effects of H_2_S-mediated S-sulfhydration modification on BraFLCs suggest a new mechanism for the modification of the FLC protein itself to regulate flowering (Fig. 7). These data provide new information that will benefit future studies on flowering regulation in plants and have opened up a new line of research on the physiological functions of the gasotransmitter H_2_S.

### Functional diversification of *BraFLC* homologs in heading Chinese cabbage

Gene duplication caused by the polyploidization of chromosomes or whole genomes may be the main mechanism underlying the generation of new genetic and phenotypic variations^29^. Overall, these paralogous genes appear to retain their original functions^30^. *Brassica* plants are thought to be ancient polyploid relatives of *Arabidopsis*^31^. The diploid *Brassica* species contain three copies of the ancestral genome. Consequently, a gene in *Arabidopsis* may have multiple homologous copies in *Brassica*, and these copies may contribute to the wide variation in *Brassica* flowering times. A total of four *BoFLC* genes have been cloned in *B. oleracea*^32^, and a previous study identified four homologous *BraFLC* genes in oilseed plants (*B. rapa*)^33^. It is not clear whether the duplicated homologs in heading Chinese cabbage have conserved or diversified functions. Here, the results showed that the four homologous heading Chinese cabbage *BraFLC* genes were typical MADS-like transcription factors. Furthermore, the binding assays of the four *BraFLC*s with eight probes from *BraSOC I* promoters showed that there were considerable differences in the types of BraFLC proteins that bound to the various probes. These results strongly suggested that *BraFLC* and *BraSOC I* homologous genes have undergone functional differentiation during the evolution of the heading Chinese cabbage. It is very likely that different BraFLCs regulate different *BraSOC I* genes under certain conditions. The complexity of this regulatory system requires in-depth genetic analysis.

## Materials and methods

### Plant material and growth conditions

Heading Chinese cabbage seeds (*B. rapa* L. syn. *B*. *campestris* L. ssp. *chinensis* Makino var. *pekinensis* (Rupr.) J. Cao et Sh. Cao) “Zhongbai 60” were provided by the Vegetable Institute of Shanxi Academy of Agricultural Sciences, China. The seeds were soaked in water at 42 °C for 2 h. Then, they were dark-cultured for 1 day in a dish to promote germination after vernalization for 20 days at 4 °C (in the vernalization experiments, the number of days had previously been determined using different experimental designs). The seedlings were transplanted to a seedling bowl and grown under long day (16 h light) or short day (10 h light) conditions at 23 °C.

### Plant material treatments

In the H_2_S treatment, after a true leaf (~7 days) had appeared on the heading Chinese cabbage seedlings, the plants were sprayed with sodium hydrosulfide (NaHS) solution at different concentrations (0, 50, 80, or 100 µmol L^−1^) once a day until the flowering stage. There were also the other two chemical treatments. The plant leaves were either sprayed with 2 mmol L^−1^ HA or 300 mg L^−1^ GA_3_ at a fixed time every day. Water was sprayed on in the control group as in the H_2_S and HA treatments. However, GA_3_ was dissolved in ethanol. Therefore, equal amounts of ethanol were sprayed as an untreated control in the GA_3_ treatment.

### Flowering-time analysis concepts

The days to budding refers to the days from transplanting to floral bud emergence; the bolting leaf number was defined as the number of leaves before bolting; the days to initial flower appearance indicates the days from transplanting to the first open flower; and the days to half flower is the days from transplanting to the first open flower appearance in half of the plants.

### Gene cloning and protein expression

All of the conventional molecular biology experimental procedures have been previously described^5,34^. The access numbers for the four heading Chinese cabbage *BraFLC* genes are Bra009055, Bra028599, Bra006051, and Bra022771, which were named *BraFLC 1*, *BraFLC 2*, *BraFLC 3*, and *BraFLC 4*, respectively. The information provided in the Brassica Database (http://brsicadb.org/brd/flowergene.php) was used by Shanghai Bioengineering Co., Ltd, China, to design and synthesize the primers (Suppl. Table 2). In addition, *Nco*I (5′-CCATGG-3′) and *Xho*I (5′-CTCGAG-3′) sites were added upstream and downstream of the primers, respectively. First-strand cDNA was generated from 1 μg of seedling RNA using an All-In-One RT MasterMix kit (Abm, Nanjing, China). The annealing temperatures for the amplification of the four *BrFLC* amplifications were 65 °C, 62 °C, 58 °C, and 62 °C. Then, the amplified genes were inserted into the pET28b vector. After they had been confirmed by sequencing, the proteins were expressed and purified using the BL21(DE3) strain^34^.

### Protein and DNA-binding assays

The 1500 bp (for the five *BraSOC I* homologs) and 2000 bp (for the five *BraFT* homologs) promoter sequence information in heading Chinese cabbage (Suppl. Table 1) was analyzed using the Brassica Database. The CArG boxes contained in the *BraSOC I* and *BraFT* promoters are shown in Suppl. Fig. 6. The probes for the binding assay were prepared after primers used to amplify the CArG box had been designed (Suppl. Table 2). The eight probes for the *BraSOC I* promoter were named pSOC I 1-1, pSOC I 1-2, pSOC I 2-1, pSOC I 2-2, pSOC I 3-1, pSOC I 3-2, pSOC I 4, and pSOC I 5. The six probes for the *BraFT* promoter were named pFT 1, pFT 2, pFT 3-1, pFT 3-2, pFT 4-1, and p pFT 4-2 (Suppl. Fig. 6). The amplified products were cloned into the pUC57 vector and acted as templates for probe amplification. All the plasmids were confirmed by sequencing.

The induced and purified BraFLC proteins (with His-Flag) were fully mixed with nickel-chelating resin (Invitrogen, Life Technologies, Inc., Carlsbad, USA) overnight. The double-stranded DNA probe was then added, and the mixture was incubated. The binding reaction was carried out according to the following reaction system: 15.0 μL of double-stranded DNA, 2.5 μL of 5 × DNA-binding buffer, and 5.0 μL of mixed purified nickel-chelating resin in a total volume of 22.5 μL. After the reaction and washing, the DNA bound to BraFLC-His was eluted by centrifugation at 500 *×* *g* for 2 min. The supernatant was absorbed. Then, the probe was amplified by PCR using 2 μL of the supernatant as a template. The binding pattern assay for BraFLC proteins with the CArG box probes in the *BraSOC I* or *BraFT* promoters was conducted as previously described^35,36^.

### Biotin-switch assay of protein S-sulfhydration

S-sulfhydration was detected by the biotin-switch method using the purified BraFLC protein treated with H_2_S. The method was performed as previously described^24^. Briefly, BraFLCs treated with H_2_S and acetone-concentrated protein were dissolved in 100 μL of HEN [250 mM Hepes-NaOH (pH 7.7), 1 mM EDTA, and 0.1 mM neocuproine] buffer, and the protein was then treated with different concentrations of NaHS and dithiothreitol for 30 min at 4 °C. The acetone-precipitated protein was dissolved in 100 μL of HEN buffer again. Then, 400 μL of methyl methanethiosulfonate blocking solution was added and the solution was left to react overnight at 4 °C. Next, the acetone precipitate was dissolved in 100 μL of 1% SDS HEN buffer, 30 μL of biotin- N-(6-(biotinamido)hexyl)-3′-(2′-pyridyldithio)-propionamide solution was added, and the mixture was left to react for 3 h at 25 °C. After the reaction, the acetone-precipitated protein was dissolved in 80 μL of HEN buffer and the solution was subjected to western blot analysis using biotin antibodies, secondary antibodies labeled with alkaline phosphatase, and nitro blue tetrazolium/5-bromo-4-chloro-3-indolyl phosphate color detection.

### Real-time PCR (qRT-PCR) analysis

After the first true leaf appeared, the heading Chinese cabbage seedlings were sprayed with 100 µmol L^−1^ NaHS solution or 2 mmol L^−1^ HA as described above. Total RNA from plants treated with H_2_S for 7 days was extracted using the TRIzol^®^ method and reverse transcribed as described in the literature^5^. The quantitative PCR primers were designed as described in Suppl. Table 2 and *BraACTIN* was used as the internal control. The expression levels of the *BraSOC I* and *BraFT* homologous genes were detected with a Bio-Rad CFX96 PCR Detection System (Bio-Rad, USA) and the relative expression levels were analyzed using the 2^−ΔΔCT^ method using three biological replicates^5^.

### Statistical analysis

The data are expressed as the mean ± SE. At least three independent experiments were performed for every test and the number of plants per biological repeat was 60 plants when the days to initial flower appearance, days to half flower, leaf bolting rate, and other indicators were measured. The statistically significant differences were analyzed by SPSS version 19.0 (SPSS, IBM, Chicago, IL, USA) using Student’s *t*-test. Different letters show that there were significant differences between samples. The probability level for significant differences was *p* < 0.05 and was *p* < 0.01 for highly significant differences.

## Supplementary information

pUC57-BraSOC Is construction

Suppl. Table 1

Suppl. Table 2

Effect of H2S on flowering in Chinese cabbage

Nucleic acid sequence alignments for BraFLCs in Chinese cabbage

Protein conserved domain prediction and phylogenetic analysis of BraFLCs in Chinese cabbage

Photographs of the pET28b-BraFLC gene constructions (A) and the purification of recombinant BraFLCs (B)

Motif analysis of the promoters for the different BraSOC I and BraFT genes

Schematic illustration of the CArG-box location in the BraSOC I and BraFT promoters for Chinese cabbage
