# Supplementary figures and images for: Hydrogen sulfide promotes flowering in heading Chinese cabbage by S-sulfhydration of BraFLCs
Source: Hortic Res. 2021 Feb 1;8:19. doi: 10.1038/s41438-020-00453-3 (PMC7848000; doi:10.1038/s41438-020-00453-3)

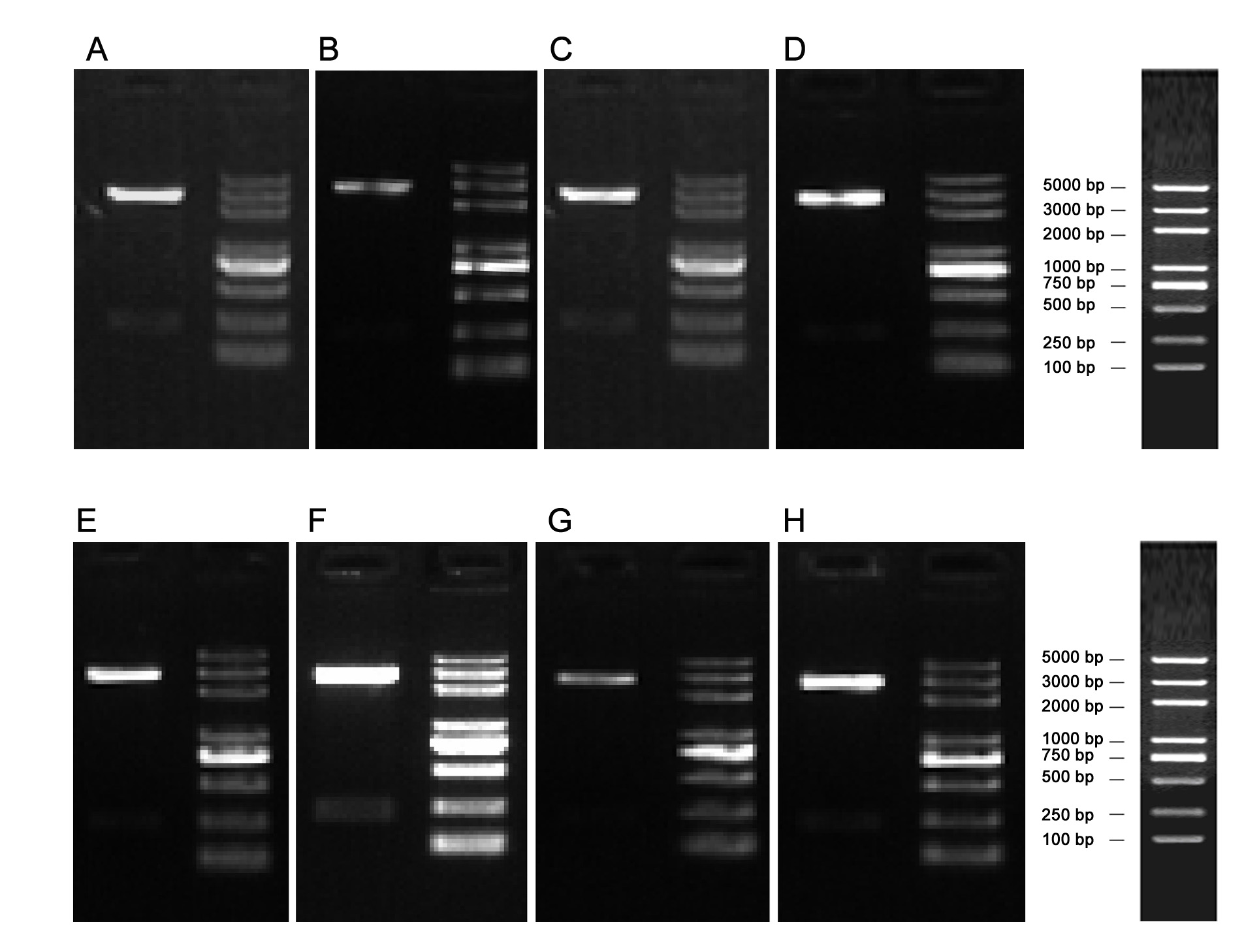

Supplement: Supplementary file 1 — pUC57-BraSOC Is construction [file 41438_2020_453_MOESM1_ESM.jpg]

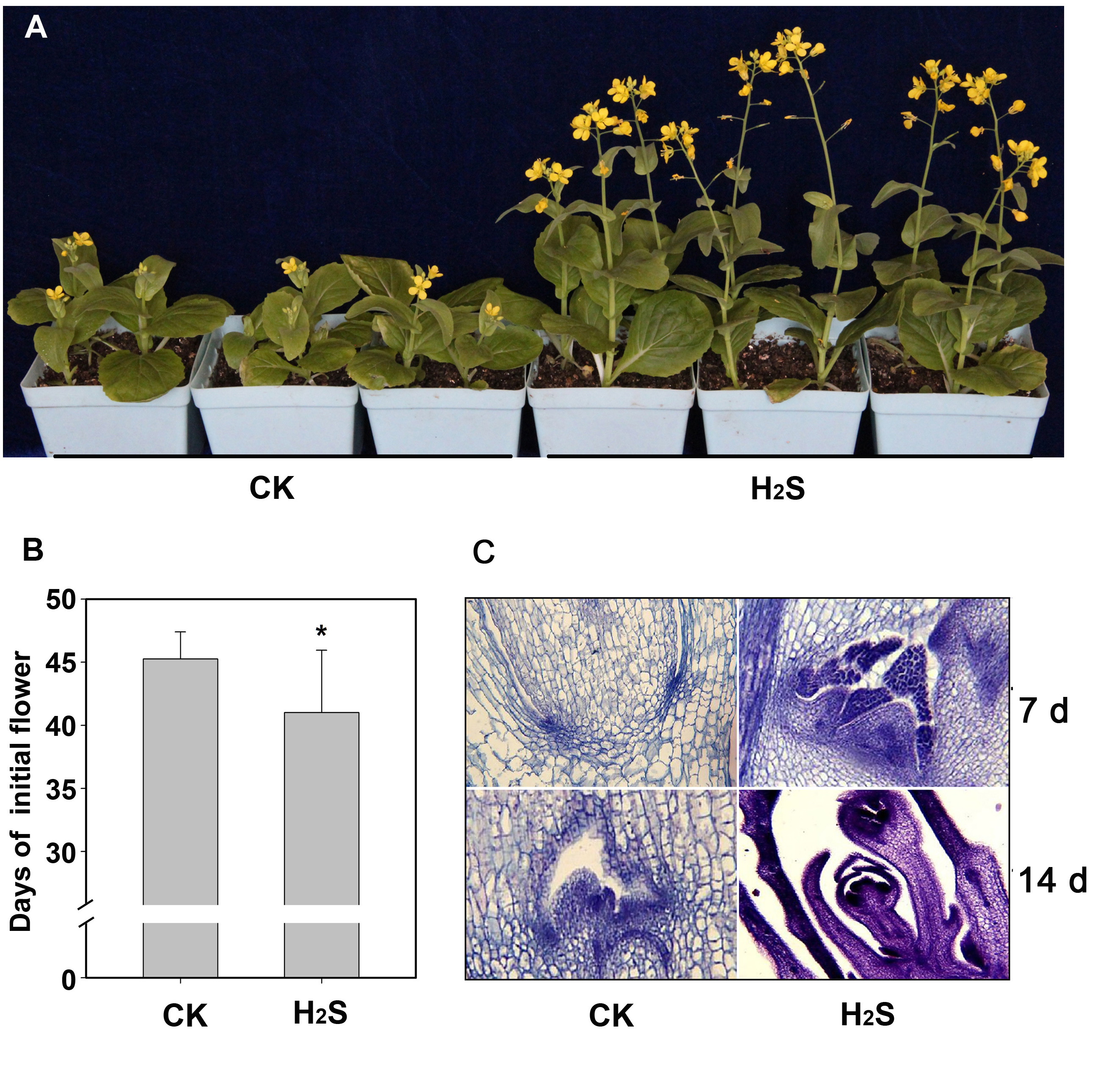

Supplement: Supplementary file 4 — Effect of H2S on flowering in Chinese cabbage [file 41438_2020_453_MOESM4_ESM.jpg]

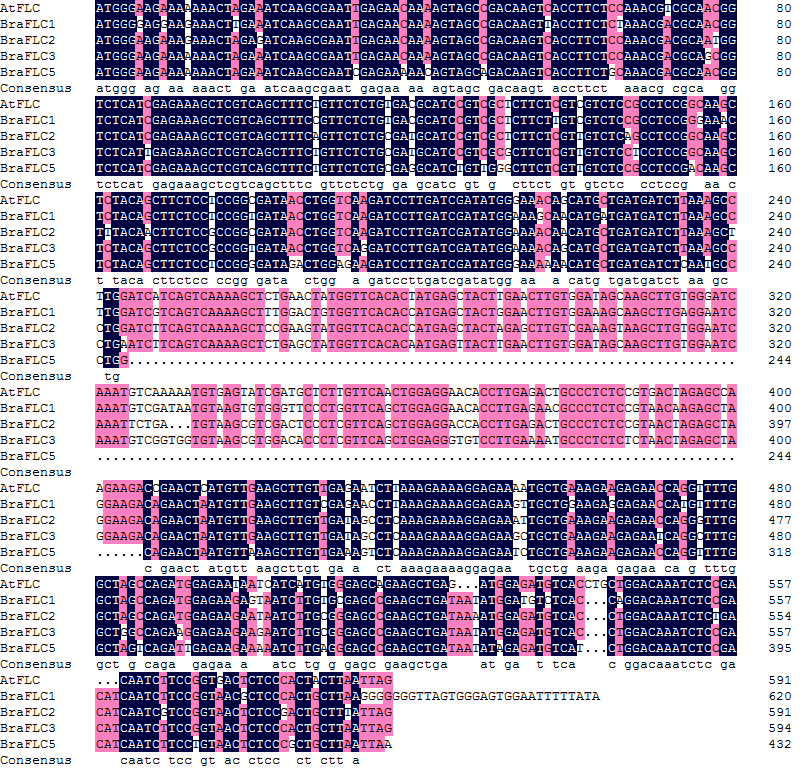

Supplement: Supplementary file 5 — Nucleic acid sequence alignments for BraFLCs in Chinese cabbage [file 41438_2020_453_MOESM5_ESM.tif]

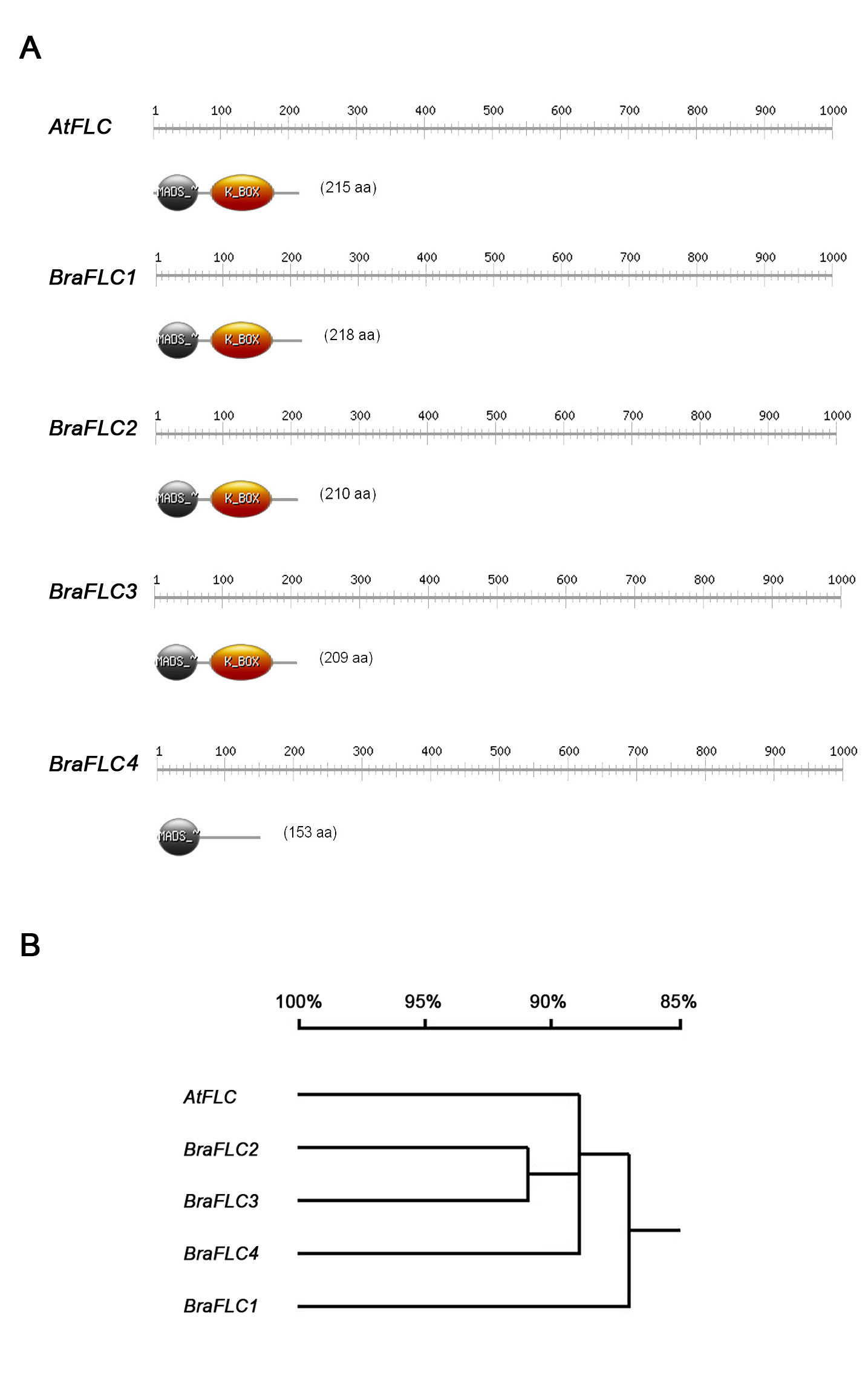

Supplement: Supplementary file 6 — Protein conserved domain prediction and phylogenetic analysis of BraFLCs in Chinese cabbage [file 41438_2020_453_MOESM6_ESM.jpg]

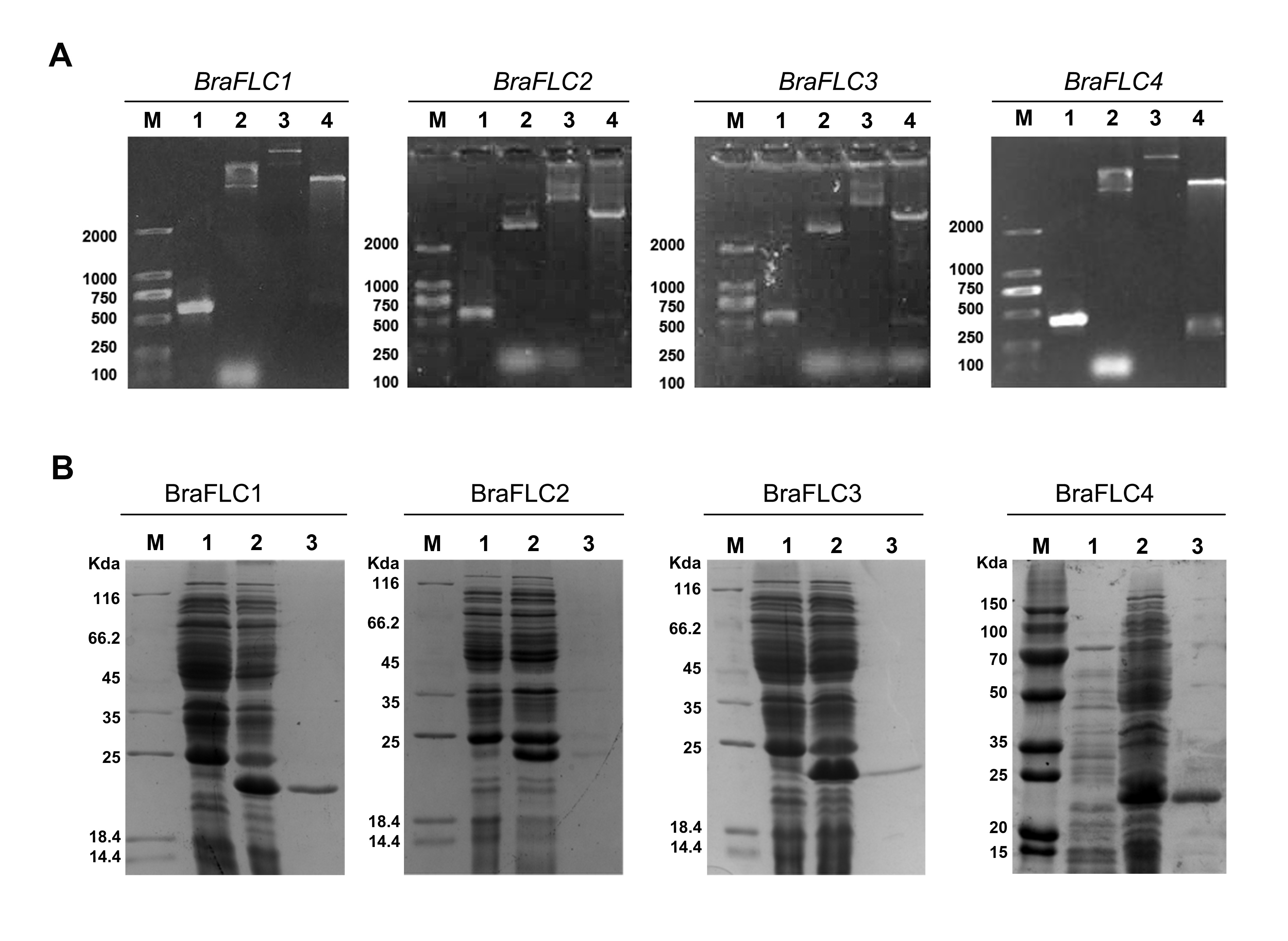

Supplement: Supplementary file 7 — Photographs of the pET28b-BraFLC gene constructions (A) and the purification of recombinant BraFLCs (B) [file 41438_2020_453_MOESM7_ESM.jpg]

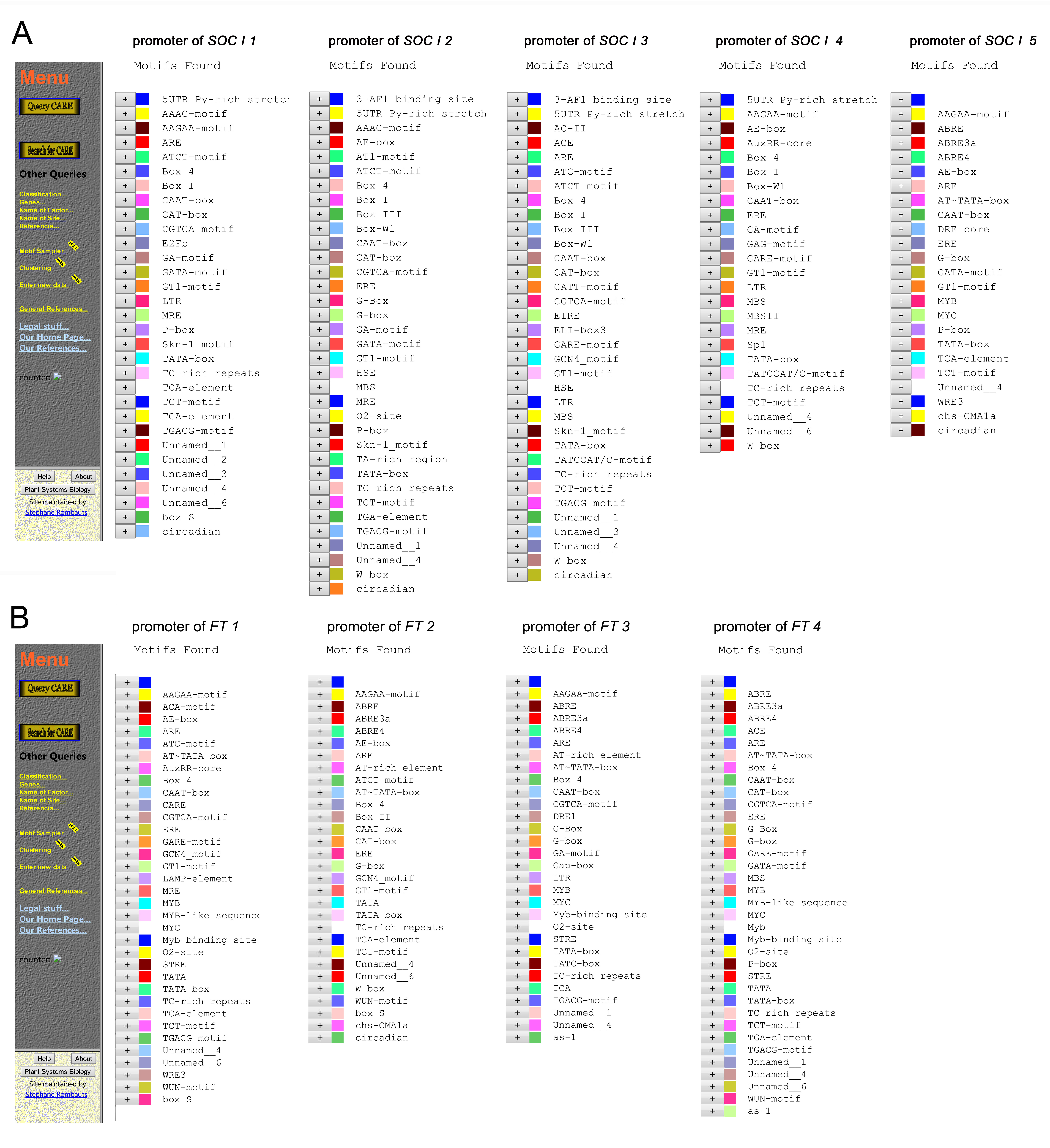

Supplement: Supplementary file 8 — Motif analysis of the promoters for the different BraSOC I and BraFT genes [file 41438_2020_453_MOESM8_ESM.jpg]

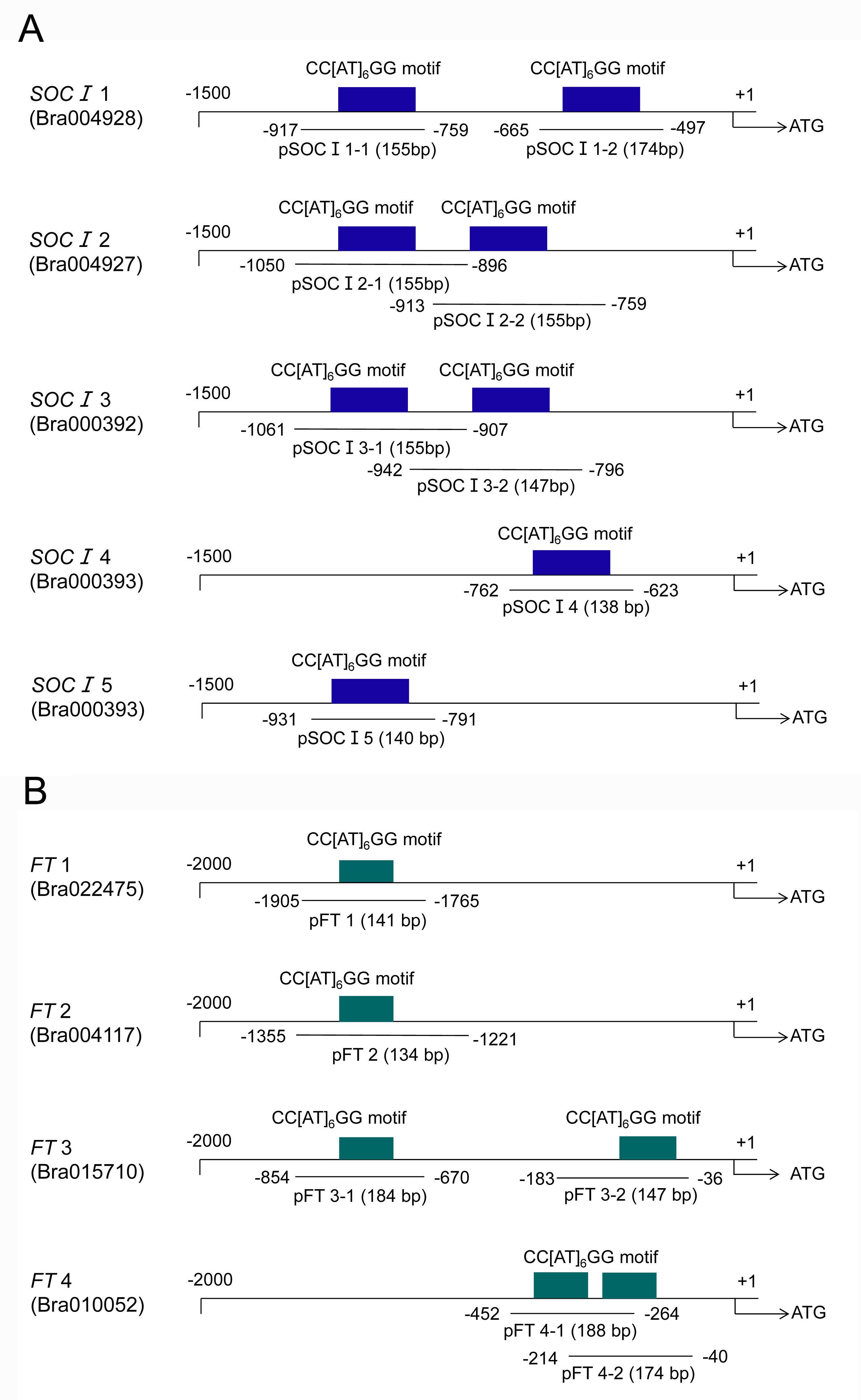

Supplement: Supplementary file 9 — Schematic illustration of the CArG-box location in the BraSOC I and BraFT promoters for Chinese cabbage [file 41438_2020_453_MOESM9_ESM.jpg]
