# Supplementary material for: Hydrogen sulfide promotes flowering in heading Chinese cabbage by S-sulfhydration of BraFLCs
Source: Hortic Res. 2021 Feb 1;8:19. doi: 10.1038/s41438-020-00453-3 (PMC7848000; doi:10.1038/s41438-020-00453-3)
Supplement: Supplementary file 2 — Suppl. Table 1 [file 41438_2020_453_MOESM2_ESM.docx]

**Suppl table 1 The sequence information of *BraFLC, BraSOC I*，*BraFT* CDS and the promoters**

| Name | Sequence |
| --- | --- |
| ***BraSOCI1***promoter | ATGATGGTCT TGTGATATAT TCATCTGCGT TATCAATAGA TCCCAAACTT TTATGATATT CATCTGCAAA  GACTAAGTAC ACAAAATATT GGATTGTTTT TTTACAGCAT AATAAACTAC TCTACTAAGG AGCCACCGGA  TCGGAAAGCC AAACCGGAGG TATGAAATCA ATATATAACA TAGCTGAAGG AGAACTCCTC GCACCTCGTG  CAAGCTTATC CGCCATTGTG TTTTGTACTC TTGATATATG TATTACTGCG GCAAAACTCC TCCATATGTG  TAGCAAAAAC TGACCAATCT TCATGTGACG ACACCATTTT CAGGTAACAC ATCATAACTC TAGCCTGTTA  TATTAGAATT ATATATATAT ATTAAATATC CTTAATTTCA GATTAATTTC ATGTGGGGGC ATATATGTAC  GAGAGGGTGT TTGTGTCCAC ACTTAAAAAA ATGATTTTAA AAATCTTATT AAACATCAAT TCTAGATATT  TATGAAGGGA AAAAGATGTG TAGGTAAATA TTTCCATATA TATCAAAATA TGCTATTTTG GTCCTTTTTG  TGGCTATTTT TCCAAAATAA GTAAAGGATG AGGTTTCAAG CGACCATCAT ATTTGCGACA CATATTACTA  GTTTCTCTTC CTTTTATGCC AATATTTTTA ATATTATTTA AATACTTTTC AAAGAAAGAA AAAGTAAAAC  GAAAATAATT GTTCCACACC ATTTACCATA ACTACAACGA GAAGAGGATC TTTTTAAGAG AAAGCAGAGA  TAGAGAGAGA AGAGAAGAGA GGAGTGTGTG TGAGTTTTGT CTCTTGTTTC CTTTATAACA CACAAATAGA  TGGAACGAGG AAAGCTACTA CTACTTCTTT TGTTACTTCC ATAAAAAGAT CCTTCCTTTC GCAGAGAATC  TAGTTTCTCT TTTCTTTTCT TCTTCTCGTA GCTCCTCCGT TAATGCTTGT ATAAGTCTCC TATATCTTCC  TATTTATACA CAATCCTGCT TCTTAGTTAA CTCTTCTATT AATCTCCCGT ACAAGCTTCG TGGTTAGGTT  TTCTATATTT ATTAACCCTT TTGGCCACTG GTATGACCTC TCCTTCATGA TCTTTCTTAC TTTTTGCTTT  ATAATCTTGG ATTAATCTGA TGGATCTGCT TTAATTTTTG TTCGATTTGA TGTTTGTATC TGGTTTTATT  ATGGTTTGTT TTGTTTCGGC TAAATCTCAA GAAAGGGTAC TGCATCTTTC AATTAATTCC AATAACAATA  TTAAAATTGC ATCCTTCATA TTCGTTTTTA AGCAATCCAT GTCACAAACT AATAGATGTG TACTTTATAT  TGATTTGCTT TTCGGGTACT TAATCATCTT CATTTCTCGT TGACTTCATC AATCAAATCC TAGGGGAAAA  CATATTTTGA TCATGAACCT CCTTTTTTGA ATGAATAGGT CGTTTTATGT GTATGACTAT AGTTTTGTTT  TGAAAGGGAA ACTAAACAAG GAGAAAAA |
| ***BraSOCI 2*** promoter | TTGTTTGTTG TTCCATGTGA TGATTCTCTG TTCGTTCGCT AATTTGTGTG CTGTTCCTCC AATAGATGGG  AGTATCTAAC AGAAGCAAAG AAACATAAGT GACGGATCCA TTGATTTTCT TAAAAATTGT GAAACCTCGG  ACAAAGGAAG AGAACTTCGT TATTTTTCTA AATCTGTGTA AAAAATGATG AGGTTTGAAT TTTTTTTTTC  TTACTAGAAG ACCATAAAGA CTGACACGTT CACTGGCATA TCAAAATTAT TACATGTGAC GACTATATTT  TAGGGAAGCT TCGCTAGCTC GTTAGCCAAA ACTAATGCGA TGTTTTTTGA CCTTTTTTAC TAAAAAAAAA  GCAAATGATG TTTTTTTTTT ACTTGAGGAG GCAAAAACTA TAGTTGAGCT GATGTGGTAG AAAATAAGTT  AAACTGTCAA TCAGGTATTA AGTTATAAAG TGAACCTTCA TCAACAAAAT AAAAACTGAT ATAAATCATT  CAGCTATTAG CATTAAAAAT TCAAATCTAA TCTTGTAAAC AAAATCAAAT TAGATTGAAT CCATATATGC  ATATAGGGGA ATATTTCAAA TCATTTATAG TGGAAATAGC GTGAGCTTCG CTTATTGTAC TTGTTTACCT  AACATCTTAC TATTATTAAC CAAATATGTG AAACCTCCAA AAACGACGAT GAATTTTCTG CGATCCTTTT  GACCAATTAA ATTTTCAAAT ATTGGTAGAA CTTAACCAGC AGTAATTTCA TACGTAATCA ACATCGTTCA  ATTTAGGTTA TATAAACATT CTAACTAAAT ATGGTAATAT ACTGAGCCAC TGCTATTTAA TTAAATTATT  TGTAACGGCG ATTACCTTTA TAAAAATTTA AGAGATCAAT GCTTCACCCG AGTAGACAAC CGATTTTTAT  GTTTAGTAGC TAATATACCA ATTAGTTATC ATGTAATAAT AAAAAAAAAT TCCTGACCGT GAACATGAAC  TATAACATTT GAAGAAAAAA ACTGAATTCG TTTTTTTTAG GCAACAAAAA ACTGATTTCG TTTGAATTTG  AAATATCAAG CTCTTTGGAA AGCAAAGAAT AATATAGTTA TTAAAAAGTG AAAATAATTG AACTTCTAGT  GGTCCAACTG TTACAAGAAG ATTTCCATTC TGAGGTATTA ACTAAAATTA CGAGAACATA GTTATCATTT  TGCTCCATCA AAATTGACAT CATTTTTAGT TTATTTATTT CTAATTTCTA TTTCTAAAAA TTTATCATTT  GTTGTACTAC TTACTTAGTA CAGTCGAGAA ACAATAATTA ATGATATTTA ATCAATCACC ATTTTAACCT  AAAAGGGTTT TACCTATTTA TATATATACA CACATATATA TATATATATA TATATATATA TATGACACGT  TATCGTGTAC GATACAATAT ATTCCTTTGA TATACTTCAA AAAGTTACTA GTTCCTGATA AGAGAAGAAA  TCAGAATCAA GAAAAAGGAG AGAGAA |
| ***BraSOCI 3*** promoter | GATTTTCATA AACAACCGAG CATATCATAT ATATCTGAAA CCAAAAATTA TTTAAAATAA ATTTATTTAA  TGGTGCAAAG ACAGCATTCT AATTTTACTA ATTAACTTAA CTCCTTTTAA TGGGTAAATA TGTGGTTTAT  AGACGAATTA AAATATAATA TTCGACCCGG ATCTGTGGTC GTAGCGGTAG CCCCAATGAC CATATATAAT  TAAGATTGAT ATATGTTCTT CATATAGTAT TCTATCATAG GTTCTACCAA CTGATGTGCT TCTACAAAAT  TCAGATTTAG TGAAAACCAA TTAATTCAAA ATCCGGTAAA ATCCAAAAAC TCGTCATTAA CCTTTGAACC  AACACCGGTT GACCCAAAAA AAAAACTTAG AAAAATATGA AGTATTGTAC ATGCATCAGT ATATAAACCG  TTTATTAGTT TAAAATATGA AGTATTATAC ATGCATCAGT AAAGATATTT TCTTTTTATC AAAACATCAG  TAAAAATTAA TAATATAGTT ATTGAATATA AAATGTGTAC CGAAAAATTC CTTCTTAGGA CATCAATTAA  TAATAATGAC ATTTACACTG GTTAATGGTT AACCGTGGAT GGGATTGTGT TTGGTACTAT CCATACATAG  ACCCAGTTAA TAATTACGCC AATCAATATA TAAACCAAAA GCCACTTATT TGGTAAATCA ATCAAAATAC  ATGGTTATTA AAATAATATA GATATTGACT GAATTATTGT TAGATAAACA TATAGTAACA TATTGTTAGT  CTAAATTTAA GGTAAGACCA AAGTATTACT AGTTTAAGGA AATGGTTCAA ACAATTTTTA TATGTAGATT  TTTTAAGACT TCTTCCCTTT ACTAGTTTAA GGATAGAGTC CAAACAATTT TTATAGGTAA ATTTTTTAGG  ATTTTTTCGC TTTTAATAGT ATTGACTTTT TTTGTCAACT AGACACAACT ACTAATATAA CAATTAGTAA  TCTTTTAGTA AAAAAAAACA ATTAGTAATC ATTTAGGCAA AAAAAAACAT TTAATGACCA TTCATGTGAA  CCATAACATT TGAAAAAAGA ATGAAGTCTT TTGGATTTGT GTATCTTTGA AAAGCAAGGA AAAAAGAAGA  AATTGCGTGA TTAAAAGAGG AAATTAGTGG AATTTTTACT GGTCCTACTG TTACAAGCAG AGTTTCATTC  TTAGGTATTT TACCTGATAC TACGAGAACA GAGTTATCAT TTTGTTCCAT CAGAAATTGA CTCATCGTTA  TTTATTTAAA ATTTCTGTTT TTTTACAATT AAAATAAAAT AAATCTCATT ATTTGGTATC ACATACTACA  GTTGGAAACA CGGATATTAG ATTATTCACC ATATTAATCT AACAACAAAT ATATAAATTA ACAAGAGTTT  ACATTTGATG TCAAAAAACA AGGGCTTACA TAAATATAAA ACGTATATTT TCGTGAGGAG AGAGGAGCTC  AGTATCAAGG AAAAAAAAGA GAGAGAAA |
| ***BraSOCI 4*** promoter | GAACCCGAAC GGGTAATACC CGAACCCGAA TGGATATCCG AAGATAACCG AACATATGTA TAATTAACCT  TATGTTTCTA GTTTATATCT CTCATTTTAT ATAAAATATT TATATTGATA CTACACATAC TTTAAGTTCA  TATGATATAC ATACAATTAC GGAAAAAAAT GTTTTGTTAC TCACTTAAAA TGCATGTCAA GTTTTTTATT  TCAAAAATTA ACAAAAAGTT ACATCCAAAA TTTTAAAAAA ATAACTAAAT TAATGTCTTT TTAGTTTTAA  AATGTTATGT CCAAATCTAT TAACCATTCA ATCTATTAAA AATAAAAAAT TAGTTAACTA AAAGTTATAT  TTTTAAATAC AATAAACTTG AGAAATGAAA ATTTTAATTT TTTTTCAAAA TCTAAATATC CGAACCCGAT  CCGAGATAAC CGAATCCGAA CTAAAAATAC CCGAACCCGA CCCGAAGTAC AAAAATACCC GAACGGGTTC  TACACCTCTA TACCGAAATA CCCGAAAATC CGAAATACCC GACCCGAACC CGAACGGGTA CCCGAACGCC  CACCCCTATT TTCGACACAT ATTACTATTT GCTCTTCCTT TTTGCTATTA TTTTAATATT ATATACTACT  TTTCAAATAA GGAAAAAATA AAAACGAAAA TAATTGTTCC ACACCATTAC CATAAGGTCC ATAACTACAA  CGAGAAGAGG ATCTTTTTAA GGAGAAAGCA GAGAGAGAGA AGAGAGTAGT GTGTGTGTGT GTTGTTGTCT  CTTGTTTCTT TTATTACACC AGATAGATGA AACGAGGAAA GCTATTACTT CTTTGGTTAC AACCATAAAC  AGATCCTTCC TTTCGCAGAG AGTCAAGTTT GATCATCTTC TTCTTTTTCT CGCAGCTCCT TCATTAATGC  TTATAAAGTC TCCTATATCT CTCTATCTAT ACACAACTGT GCTAATCAGT TTCTACTTCT ATTAATCTTC  GTGATTAGGT TTTTTATTTA TCAACCCTAT TGACCACCGG TTAGATCTCT TCGTCTTGAC CTTTCTTTAC  TTTTTGCTTT GTAGTCTTGG ACTAGTCTGA TGAATCTGCT TTCATTTTGT TGGATTTGAT GTTTTGTTTC  GGCTAAATCT CATTTCAATT AAAACCCCCC TAAAAATCAA TATTTTTTAT AAAAGGTTGT ATCTTTCATT  CATTTTTAAG CACAAACTTG TAGATCATAC AAGTTTGTAC TTTACATTGA TTTCCTTTTT GGGTACTTAA  TCATGTTCAT CTCTCGTTGA CTTCATCAGT CTCCTCCCCC CAAAAATATA TATCAAAAAT CAAACCCCAG  GAAAATAATT CTTATTTCTA TCACGGGTTA AATTATGTGT AAGACTAGTC TTGTTATCGA AGTGGTTGCA  GTTGCACCAT TGATCTCATG TTTTCTGCAA TAAACCCAAA GGAAGATTGT ATAAATATGT TTTTTTTCTG  TTAAAGGGAA ACTAATCAAG GAGAAAGA |
| ***BraSOCI 5*** promoter | GGACAGCCAA TCCCATCAAT ATACATATTT TGAAGGGAAA AAGATGTGTG TGTAAAAATA TCCATATATA  TCAAATATGC TTTCTTGGTC TTTCTCAAGA CTTTTTTTCC AAAATAAGTA AAGGATGAGG TTTGAAGCAA  CCATCATATT TGCGACACAT ATTACTATTT GCTCAGCCTT TTTTGCTATT ATTCTAATAT ATATTTAATA  CTTTTCAAAA AGAAAAAAAA ATGTTCTACA TTATTATTTT TTAGTTCGGG GTATCTCCCA CTTAAGATAA  GTGAAACTCA CGTATATAAT TTACTTCTGC ATGTTCAAAC GAGGCCTAAA TCATAACATT ATATCCACAT  GGTCACACAT ATTTGGTATT GTGGAATTGA TTTGAACTCG AATCTTTTAG GACATTTTCG ACCATACTCT  ATTTTTTTAC TCTAAAATAG AATGAAAGTG AATATGAAAT AAAAAATACT CTAATCTTAC TTTATTTTTT  TATTCCATAA TGTAGTTTAT TTCATAAGTA AAGTGATTTA TATTTTGTTT GTTCATTGCT CAATTATAGA  ATAAAAATAG AGTAGAATTA AAACATTTTT ATTCCTTATT TTTTAACTCT ATTTTAAAAG AAAAAAATAG  AATTTTACAT TGAAAATACT CTTACTTCTC AAAGTCGGCA TACCGCTAAA CCAAACCAAC CATGGTTGTT  CCCTATTACC ATAACTACAT CTTGAAGAGA GGAGAGTGTG CGAGTCTTGT CTTTTGTTTC TTTTATTACA  CACAAATAGG TGAAACGAGG AAAGCTACTA CTACTTCTTT TGGTACTTCC ATAAATAGGT CTTTCCATAC  GCAGATAATC AAGTTTGTTC ATCATCTTCT CGTCGCCTCT CCATCAATAC TTATATCTAT CTATTTATAC  ATAATCGTGT TACTTAATTT CCTCTTCCGT TAATCTCCTT AAAACCTTTT AGGGTTTTAA ATCCTTTTGG  CCACAGGTAG ATCTCTTTGT AATGATCTTT TCTACTTTTT GATTAGTCTG ATGGATCTTT TTCATTTTGG  TCGATTTGAT GTTTTGTTTC TGGGTTTTTT TTTTGTTTTG CTTTGTTTAG GCTAAATCTG ATGAAAGGTT  GCATATCTTT CAATTAACCC CCATAACAAA ATAAAATAAA GGCTGCATAT TTCATTCATT TTAAGCAATC  CATATAGATC AACTATATAA ATTTGTACTT TAGATTGGTT TGGTTTTTGG GTACTTAATC ATCTTATCTC  TCGTTGACTT CATCAGTCTC CTTCCACCAA AAAAAAATCC CAGCAAAAAG TTTATTTTTT CTTTTCCAAA  TGGATTGTTT TATGTGTGTG ATTTTTTTTT TCCAAATGGA TTGTTTTATG TGAGTGAATA ATCTTGTTAT  TGAGGTGGTT TCACCCTTGA TCTTATGTTC TCTGCAAGTT AAAAAGAGAT CTTATATAAT TTTTTTCTGT  GAAAGAAAAA ATTAAACAAG GAGTAAGA |
| ***BraFT 1*** promoter | ATTTTAGGAG TAATAACAGG AACGAATAAG ATCAATATAC ACGCGTCATT GAATAACCCC CTCAATTGTT  AGGTTATGGT TGTGAATTCA TCTTCGAGGA GATCAATAGA TGCTCTGAAT AGTTGAAATG GACCAATGGT  ATATATATCC ATTGTCAAAA AAGTAATCTC CCAAATTAGC AAGTTTGTTC ATAGACGCAG GCATCATCTG  GTGAGAACAA ATGGATGGTT GATATTTCAT AATTTATGGA TATCAAAATT CAGAAGTGGA CATCTTTTTT  TTTTGTCAAC AGAAGTAGAC ATCAGCTTTT AACTTTTAGT AGTTAAAATA ACTTAACAGT AATAATATTT  TTTATTTAAA AATATTCTCT GTTTATTGGT TTCAATGGTA GATTGAAATA TGATGATATG TTTATTCTTT  CTATCTCAAG TCACCTCACA GATAAATATG CCATATGTGT GTCTGCTGCT TTTGCAACTT GGAATCAAGA  AGACGAAGTT CGCCAAAAAA AACTTGGAAT TAAGAACGCT AAATCACATT TTACAACCTG CTTCTCTCGC  TATAAGTCAT TATAGGTAAC ATTCTAAAAA AAAGTCACTA TATGTAACGA TTATTCCATG TTAAAACATG  AATGCTATAC CTAAGCACTT TTTAAACGTA AGCTATATAT ATTGTATTCA TATTTTCCGA TCAAAAATAT  ATTCTACAAC AAATAAATGG CCTAAGTGTA ATTTTGATTA TTATTGAATA CCATTTGAAA TACACATATC  AAAATACTAT CAAAACATAT TTTCTTTAAA AATAAAATTA CGCCACATGT TATATTACTA AGAATGTTCT  TTAAATAATT TTAATCAATT CATAAAAGAT ACTTTACATA ATATGTGTTT TAAAATATAA AATAATCACA  TGTAATAATT ATTATATTAA TTAGCAAAAT TTTCTTATAT TAATCATTAA TATATATCTT CTTTTATATC  TTTATTTTTT TTTGTCATCC ATCTTTATCA TTTATTTTAA TTTTAAAATC AGAATATCTA CATCTTGCAT  ATAATAAAAT TTAAATTATG AGAAACAAAC ATAAATAAGA AACCATAAGT AAAGTATATT ACTTGACTGG  CATTCAAATT TAGATGTGTC GATTATCAAG TTTTATAAAT GTAAGAAAAA TTGAAAAAAT GAATTTATTT  GGATTATACA AACACTATGA GATGGGTTAA ACATGCGGAC TACCTAATTT GACTATATAT AGCTATAATT  TGACTCTCGT GAATATAACT ATAATCTTCT AGTTCTCAAA AGTAATAATA TATAATATAT TTATATACAG  TAAATACAAT TTATTATAAA ATATCGAAAA TAAATATAGT TTACCCAATA AAATAACATA TATTGTTCCA  CATAACAATA CAATATCACT TCGTTTATAT CACATATATC TTACAATGGT TATTTTATTA AAGTTAAACA  TCATAATATT ATTTAAAAGT TATGATCACC GATCCGAACC AAGTTGTGCA ATACTTCCTC CATATTAAAG  GAATGATGTT ATATATAGTT TTGTTGAGTA TTTTTTTGCA CTAATTTGCG AAAAATAACA TACTTGTAAA  GTATTTAGAG TATATGGTCG AGAGAGGTCT TTTTCTATTG GATTACAAGT CGGAGACGTA GTTAGTTGAA  AGAAATGACT CAAATTTTAT TGGCTACCAA GTGGGTGAGA TATAATTTGG AATATTATCA GTCTAGCTAC  TAGAGTGGTG GCTTTGGAAA AATACCACAA ACATAAAAAA AAAAAAAAGA CAATATGTGA TGTACATAGA  ATCAGTTTTA GATTCTCGTA CATCACTAGA CAAGAAATAT ATTGTGGTGA TGAGTTCACC GACCCGAGCT  AATTCAACTC CCAAGACACT ATATATATCA CTTACTAGTA GAGGGTTAGT TCACAAGAAC GAAGAATAAA  AAAATTAAAT CAGAAACCAC CTGTTTGTTT TAGATCAAAG ATG |
| ***BraFT 2*** promoter | TTAGGCATGG GCACTGTTAC TTGATACTCG GCTTATATTT ACTACTTTGA CTCGGTTCGG TTTAAAAAAA  CAAGTACTTG CTACAATCAA GTCGAGTAAC AAATAAACGG ATTTTATTAC TCATAAGTAC AAGTCAAATA  ACAAGTATAT TTTTATTTTT AGCTACTTAA CTTGTTACTT AGTATCCTCT ACTTGCTCCT TTACTTGTTA  TATATTATTT AGTTTATTAA ATATTTGGTA TCTAATTTAT TAGTTAATTA AAATTTGATG TATTATATGG  CTATAAAACT AAAAGAATTG TGTGTTTAAA TATAAATACC CAACATTACT AAACTATTGG GCTAGTACTA  ACCGATTTAG CCTAACGGAA ATCTGAGATT TATATACAGA GTTGTAAAAG TAAAATTATG TGTTTTAACT  TCAAGTAGTT AAGGAAAATA GGTTAAGTAA TTGAAAATAT CAAGTATCAA ATCAAGTCAA GTAAAACTGT  AAAAGATAAA ATCAAGTAAT AAACCAAGTT AAGTAATTTA TAAGTATTAA AAAAAATTGC AAGTACTTGG  TATAGCGAGT ACTTGTTCAA AATAAGTCAT ATACAAGCAA CAAATACAAG AACTTGAACA AACCAAGTCG  GGTAACAAGT ACCTAAAAAA TGCCGGGTAC TTGACCCGTG CGGCACGGGC CGTGCCCAGG CCTACCAAAT  ATGCCATATG TGTGTCTATT ATTGGAACTT GAAATTAAGA ACGCTAGCTA TATCACATTT TACAACTCGC  TTCTCTCGTT ATTTCTTAAG TCTTATTTTG AGCTACACTG TAAGAAAATA AAGCTGTCAT ATTATTTAGT  CCTTTAATAC GTGATATCTT TTTTTTCCCA TGCATTATAT CGTTTATAAA TAAACAAAAA AAATTAACTT  AAATGTTACA GCGAAACACT ATTGACATCG TATTAGATTT ATATTTTCCG ATCATGTTAT GACAAAAAGA  CCTAAAAGTA AATTTTAATA TTATAAAATT TAATTTCTAT TTTAAATTAT ATATATATAT ATATAAGATT  CCAGAAGTCC AAATTGGAGA TACAAATATC AAAAGTAAGA TAAATTATTG TGTGCTAATT AATTTATTTA  TCTTGCGTTG TTTCTGTCTA AGGTCCGGAA CACCTCTGGG TTAAATATAT ATATAACAAC TCTTCTAATA  AGAAAGTCTT TCAAAATAAG GTAGGCCACT CTTCTAAGTT CTAACGTAGA TACAATTTTT ACCAATTCGA  AAAAGTTTGT TGCAAAAGCT AAAACAAAAA CAAAAAGGAA AAGCTAATAC CATTATTTGT ATAATCAATG  TATAGCCTAA AAATAAAAAG TTATTATAGT TAAGTTAGTT ATAAATATTT ATATTTGTGT TCATTTACAG  GTAAATCGTT TGGTACAAAT TCTTCAAATC AAGACAAGTT AATTATCATT ATTACACATC ATAATTATTA  CCACAAATCA TTTAATACTC GTTCATAAAT GGGTATTTTT ATACGAAAAT ACTGTTCTTC TAAACTAGAA  AAAGTTATGT TTTCTAACAC AGATTTCGCT GTTATTTGCA CTCTTTTATG TATAAACAAT ACACTAGTAA  ATATGGTGGA GAGATATTTT ATTTATTTTG AGTCATAAGT CGAAAGGTAT ATATATAGTT AAAGGATATA  TAAAAATATT ATTGGCTACC AAGTGGGTGC GAGATAATCT GGAATATTAT CAGTCTAGCT ATTAGAGTGG  TGGCTTTGGA ATACATAAAA GAAAGAAAAT AATAATTATG GAATAAGAAG ACAATATGTG ATGTACGTAG  AATCAGTTTT GGATTCTCGT ACATCACTGT ACAGGAAAAA GATTGCGGTG ATGAGCTCTC CGACCCGAGT  TAAACCGACT CCCAAGACAC TATATATATA CGTGACATGT AGAGGGTTGG TTCAGAAGAT AGAAGAACCA  ATAAATAAAT CAGAAACAAC CTGTTTGTTT TAGATCAGAG ATG |
| ***BraFT 3*** promoter | TTAAAAACTT GGCTAATTTT TTTTATTTTG CATAGGGCAT CAAAAACTCT TTGCACGGGT CACTTACACT  AAAAATAAAA TAAAATGAAC TGACAAGCAC CAAACAAAAA TCAAATGAAG CAAATGAAAA AATTCAGTAA  ACAGGAAAAA TGTAAATAAA TGTTGTAGAT AAAAATCAAA TAAGTTTTGC ATTTTTATTC TAGTTCCTAA  AAGAATTTCC TAGTTTTAAT ATGACATGAA CAAACTAATC AACCAAACTG AAACGAAGCT TTGAATTCCT  AGACCTAGTT TAACGTGAGA GATATCAAAG TTGATGAAGG GCATATTAAG ATTCTTTTTA AGTTTCGCGC  TCATATATTC GATGAACAAT CAACTTGCTA ATGAATTAGT AATATGTCAT ATTGGAGTTG GAGAAATAAT  TATCATATAC TTATATAGGA TTAATCTAAT TATTGTCTAT TAATTGTCCA GGGACCAGGG AACAAAGTTA  TGTCGAGTGC AAAACTAAAC GTTTCGTCTC TAATCGATGA CATTTTTGAG CGATATAGTT TACTGATGCT  ATTATTGGGC TTCTTAGAAA TAGATAAACA GAAGACGTGG AAGAGATTAG GAAAGTTTGG TTCCCTCAGA  TACACATTAC CTATACATAG TAGTACGTGC CTTCTTCAGC AACTACAAGA AAAAAAATGC ACAATGCATA  TAGCATGGTT GACTGATGTA TTTTAATCCC AAAAAGAAGT AAACAGAACC AAATATTATT GGCTAGCGAG  TAGCAAGTTG GTGACATATT ATTGGGAACA TTGTAGCTTT TAATTTGAGT GGTGGATTTG GGATACAAGT  ATACAACATC ACAACCAAGA GAAAAAAGGA AAAAATGAAT AGGAAGACGA TAACTCAAGA AGTGATGTAC  GTACGTCGAA CCAGTTTTAG ATTCTTGAAG ATGACGAGAC ACGAACTGCA GTAATGATAT TTTTCAGCGA  CTCGAATATG TTAACGCCAT TCCCAAAACA TTATAAATAT AGAAATAGGA ACATAGGGTT TACACGAAGA  AAACAAAAAC CAACATTTTG TTGAGATTTG AAATCAAAGA TCATGTCATT AAGTCCGAGT GAGAAGTTCT  GTAATCATAT AATCGTTCTT ATCTTTCATC TAAGACCTCA AGGCCTATAT TTATACACAT CAGATTTCCT  AATACTATCG AATAGATGAA ACTACAATAA TGGAAATATT CTATTCTAAT GTCATCTAGG TTTGGGATAT  GTTAATATCC CAATACCTCC CCTCAAGTTG GAGCATACAA ATTTTGTATG TCCAACTTGA ACTACAAGTC  TTCAAATTCA AATAGGTTGT TGGTTCTTTC GTCAAAATAA TCAGTTCGTC GGTTTCGGTT TATCGGGTTC  GGTTTCGTCA GTTTCATCAA TTCGAGGTCA TTGTAACAAC AGGAAAATTC ACCATCCTTG TTGATCAAAG  AAAATCGCAG GTCGATAGTT AGAGAAGTTG CAAGTCGAGA GATCGATTGT TCGAGGTTTC AGGTCGTAAG  GTTGAGAGTT CGAGGTCGCA TGTCGTGAGA TCGAAAGTCG TGGGGTCGAA GTTGTTCGAT GGCCGTGAGT  GAATTCACAT GTTTGTTAGG TCGACGTTTC ATTATTCCAT GCCTTCACGT CATCATCCGG ATCGTCGTCT  TCATCTTCTT CAATTGTTTC CACCATGACC AAACTCTTCT AATTACCAGA TACGGAAGCG CTATTATGGT  CCACAAAAAT CGTTATGGCT CTGATACCAT GAGAAGTTCT ATAATCATAT AATTGTTCTT GTTATTCATC  TAAGACCTCA AGGCCTATAT TTATACACAT CAGATTTCCT AATACTATCG AATAGATGAA ACTACAATAA  TGGAAATATT CTATTCTAAT GTCATCTAGG TTTGGGATAT GTTAATATCC CAATACCGAG AGACCCTCTT  ATCGTAGGAA GAGTTGTAGG AGATGTTGTT GATCCTTTCA TG |
| ***BraFT 4*** promoter | AATGATAAAT CTTTTTAGAA ACGTTAGGGA ATGCATTATT CTTCGTCGTT TTTTAGTCACCGTTCATACC  CTAGTTTTTT ATGAAAATAT TTTAAACTTG TACATTGTAT ATTAATATTA AATGTTCTAA CGACACGAAT  GAAATTTCTT ATATACTGTT TGAGTTTGGC GAAAAGTGAT ATGTTAGCCC CTATCAAATC ATCGACGTGT  TAAATTCCAT GCAGACACAA AATAAGTTAT TTACTTTGTC AACTAGAATC TTGGTGGTTG GCTCTTACGT  TATCACTTTT TTTTGCAAGT GATCTTTAGT TTGTTAATTT ATTTCTAACT GAACAGCTCA ATTTATTATC  ACTATTAAGT TAATGTGAAC GTTTAATCTT GAACGCTCAA TCCCAATTAG GATAGATTGA TTCGTTAAAT  ATAAGATAAT ATGTGTTTAT CGGATGAATT GATTTTTCGC TGCCAAAATG AAAAGTATCA ACGAATTCTG  TTTTTTTTTG GTAAAATCAA CGAATTCTGT TTTTTTTTGG TAAAATCAAC GAATTCTGTT TTTTTTTTTT  TTTTGAACAT AACGAATTCT GTTTTGTGTT TGTGGGTTGT GTTTTTCTCT TCTTAGTTTG TTCTTACACA  AATAAGTGAC ACTATATATC GATTAGATGA TACAGTTGAA CATATATACA CATTTTTAGC CAATAATATA  CATTGTATTA AGTATATATG AAACTGAATG AAGACACAAT AACAAGTTAT TTCGAAGATT ACATATTTTC  GACAACTAGA CATTGTAATA CAGAAAAGGC AACACGTAAG GACAGCAACA GAACAAACAC ACTAAACATG  TCTTTATTCA TTTATAGTAA TTATAACCAT ATCGATAAAC AAAATAGAAA TATATAAATA AGCATTAATA  TTAGGTGCCA CTGTGTTTGC GCCACGTATT GGATTGAGCA TATGCATATA GTGGATTCCG TGACTAGAAA  CTACAAAGTG ACGAGAGATT CTCAGTCTTA TTATAAAGCA GATTCTTTTC TTTGATCTCT TCTTCTTCTT  CTTCTTATTT GGAATATCTG CAAAATCAAT TTCTGTGCCA ATCATCATAA TATCTGGCTT TCATTTATTA  TCGACACGCG TGTGTATTCT TTTTCTATTT CACCAGTCGA ATATTCGAAT GTAGTGGGGA CAAAAATTAT  TGACTATTAT CTTGTTTTTA TTTTGAATAA AATTAAATGA CAATATGTTT AGCTACTAAA CCTGGACATA  ATTCTGATTC TGTATGTTTC TACATTTAGC TAACACTATG CTGGCTACTA AACGTGGATA AACTAGCTAG  TGTGATCGTT GTTTTAAGCA TACATGTTAC ATTTCTTTTT GAAAGCACAT AAATGTTACG TTTAGCAATT  AAATCCATCA AATGTAGGTG ATCCTGTTGG AGAGTTCACT CTAAATATAT TTTGTTAGAG TTCGAATATT  TCATTATAAC TCCAGTAATT TTTGATGTGT GTCATGTAGA CGTTTCCAAT AGCAAGAAAA CATAACATGG  CTTTCTATGG ATTATGGAAT GTTAGATTTC GACCCAAAAA AAATTAACAA AAATTTGAAG TACTAGTTTC  TATAGATATA ATTATTAAAA CCAATCCACT GTTGAAATAA AACAAAAAAT ATGCATGTAA GAAAAATTAT  AAAATCATAA GTTGACCACT TAATAGAATT TTAAGTTCTA GAGATAGAAG AATCACATGA TAGATATTTA  CATATCTATT GATGACAATG CCAAATAGTA AGAATAGTGC TAGAGCATTA AGATAAACAC TCTGACTTAG  TAGAATATAT ATACATACAA AAATCCCTTT TTATCTCCCA AGTTGTGCCA AGTCCCAAGA TTCTTTAGTT  CATCTATAAA AAGCTTTCAT ATGGCTCATT CCAAAGAAAC CAGCATCAAC CACAACACAC ACTACACCAT  TGAGAAAAGT TGTAGCAACA AAAGGAAAAA GAGGTCAATA ATG |
| ***BraSOC 1-1*** CDS | ATGGTGAGGG GGAAAACTCA GATGAAGCGA ATAGAGAATG CAACAAGCAG ACAAGTGACT TTCTCTAAGC GAAGGAATGG TTTGCTGAAA AAAGCCTTTG AGCTCTCAGT TCTTTGTGAT GCTGAAGTTT CTCTGATCAT CTTCTCTCCT AAGGCAAAAC TTTATGAATT TGCCAGCTCC AATATGCAAG ATACCATAGA TCGTTATCTG AGGCATACCA AGGATCGTGT CAGCACCAAA CCTGTTTCTG AAGAAAATTT GCAGCATTTG AAACATGAAG CAGCAAACAT GATGAAGAAA ATTGAACAAC TTGAAGCTTC CAAACGTAAA CTCTTGGGAG AAGGCATAGG ATCATGTTCG ATAGAGGAGC TGCAGCAAAT TGAGCAACAA CTTGAGAAAA GTGTCAAATG TATCCGAGCA AGAAAGACTC AAGTGTTTAA GGAACAAATT GAGCAGCTCA AGCAAAAGGA GAAAGCTCTA GCTGCAGAAA ACAAGAAGCT CACTGAAAAG TGGGGATCTC ATGAAATCGA AGTCTGGTCG AATAAGAACC AAGAAAGTGG  AAAAGGTGAC GAAGAGAGTA GCCCAAGTTC TGAAGTAGAG ACAGAGTTGT TCATTGGGTT ACCTTGTTCT  TCAAGAAAGT GA |
| ***BraSOC 1-2*** CDS | ATGGGAAGAG GGAGAGTGGA GATGAAGAGG ATAGAGAACA AAATCAATAG GCAAGTGACC TTCTCAAAAA GAAGAAATGG TTTGTTGAAG AAAGCTTATG AGCTCTCTGT TCTCTGTGAT GCTGAAGTTG CTCTCATCGT CTTCTCTAGC CGTGGCAAGC TCTACGAGTT CGGCAGTGTC GGGCTTTATC TTGTGGGTAT TCTTAAAGGT TGTGTCTTTT ATCCTAAGAA CTGGTGTCAG GAGGTGGCAA AGCTGAAAGC CAAATACGAA TCGCTTGTTC GCACTAATAG GCATTTGCTT GGAGAAGATA TTGGAGAAAT GGGCGTGAAG CAACTGCAAG CGTTGGAGCG GCAGCTGGAA GCAGCTCTTA CTGCGACTCG ACAACGGAAG ACACAAGTTA TGATGGAAGA AATGGAAGAT CTTCGGAAAA AGGAGCGGCA ACTCGGAGAC ATAAACAAAC AACTCAAGAT TAAGTTTGAA GCCGGAGGCC ATGCTTTCAA ATCCTTTCAA GACTTCTGGC CAAACTCGGC AGCATCGATG ATGGCCGGTG ATCCTAACAA TTCTAAATTT CCGGTTCAGC CTTCTCATCC TGATTCAGTG GATCGCAACA CCGAACCCTT TTTACAAATA GGGTTCCAAC AACATTACTA CGTGCAAGGT GAAGGGTCTT CGGTACCAAA GAGTAATGTG GCATGTGAGA CTAATTTCGT CCAAGATTGG GTTCTTTGA |
| ***BraSOC 1-3*** CDS | ATGGGAAGAG GGAGAGTGGA GATGAAGCTA ATAGAGAACA AAATCAATAG ACAAGTGACC TTCTCAAAAA GAAGAAACGG TTTGATGAAG AAAGCTTATG AGCTTTCTGT TCTATGCGAT GCTGAAGTTG CTCTCATCGT CTTCTCTAGC CGCGGAAAGC TCTACGAGTT CGGCAGTGTC GGAGTTGAAA GAACAATTGA ACGGTATCAT CGTTGCTACA ACCGTTCTCT GAGCAATAGT AGGCCTGAGG AGTCTACACA GGCATGTAAT TGGTGTCAGG AGGTGACAAA GCTCAAATCC CATTACGAAT CTCTTGTTCG CACTAATAGG CATTTGCTTG GAGAAGATCT TGGAAAAATG AGCTTGAAGG AACTGCTAGG GTTGGAGAGG CAGCTGGAAG CCGCTCTTAC TACGACTAGA AAACGCAAGA CACAAGTTAT GATAGAAGAA ATGGAAGATC TTCGGAAAAA GGCAAGTTAT ATAATTGAAA GGCAACTCGG AGACATAAAC AAACAACTCA AGATTAAGTT TGATCAGGCC GAAGGCCTTG CTTTCAAATC GTTTCAATAT TTATGGCCAA ACACGGCAGC ATCAGTGGCC GGTGATCCCA GCAATTCTGA ATTTCCGGTT CAGTCTAGTT CAGTGGATTG CAACACCGAA CCCTTTTTAC AAATAGGGTT CCAACAACAT TACTACGTGC AAGGTGAAGG TTCTTCTGTA TCAAAGAGTA ACATAGCATG TAAGACCAAT TTCGTCCAAG ATTGGGTTCT TTGA |
| ***BraSOC 1-4*** CDS | ATGGTGAGGG GAAAAACTCA GATGAAGCGA ATAGAGAATG CAACAAGCAG ACAAGTCACT TTCTCTAAAC GAAGGAATGG TTTGTTGAAG AAAGCTTTTG AGCTCTCAGT GCTTTGTGAT GCTGAAGTTT CTCTGATCAT CTTCTCTCCT TCGTTATCTG AGGCACACCA AGGATCGAGT CAGCAGCAAA CCGGTTTCGG AAGAAAATAT GCAGCATTTC AAACATGAAG CAGCAAACAT GATGAAGAAA ATTGAACAAC TTGAAGCGTC CAAACGTAAA CTCTTGGGAG AAGGCATTGG ATCATGCTCG ATTGAGGAGC TGCAGCAAAT TGAGCAACAA CTCGAGAAAA GTGTCAAATG TGTTAGAGCA AGAAAGACTC AAGTGTTTAA GGAACAAATT GTGCAGCTCA AGCAGAAGGA GAAAGCTCTA GCTGCAGAAA ACGAGAAACT CGCTGAAAAG TGGGGATCTC ATGAAATCGA AGTTTGGTCG AATAAGAACC AAGAAAGTGG ACGAGGTGAC GAGGACAGTA GCCCAAGTTC TGAAGTAGAG ACACAATTGT TCATTGGGTT ACCTTGTTCT TCAAGAAAGT GA |
| ***BraSOC 1-5*** CDS | ATGGTGAGGG GAAAAACTCA GATGAAGAGG ATAGAGAATG CAACAAGCAG ACAAGTGACT TTCTCTAAAC GCAGGAATGG TTTGTTGAAG AAAGCCTTTG AGCTCTCAGT GCTTTGTGAT GCGGAAGTTT CTCTGATCAT CTTCTCTCCT AAGGGAAAAC TTTATGAATT CGCCAGCTCC AATATGCAAG ATACCATAGA TCGTTATCTG ACGCATACCA AGGATCGAAT CAGCAACAAA CCGGTTTCTG AAGAAAATAT GCAGCATTTG AAACATGAAG CAGCAAACAT GATGAAGAAA ATTGAACAAC TTGAAGCTTC CAAACGTAAA CTCTTGGGAG AAGGCATAGG ATCATGCTCG ATTGAGGAGC TGCAGCAAAT TGAGACCCAA CTTGAGAAAA GTGTCAAATG CATTCGAGCA AGAAAGACTC AACTGTTTAA GGAACAAATT GAGCAGCTCA AGCAAAAGGA GAAAGCTCTA GCTGCAGAAA ACCAGAAGCT CACTGAAAAG TGGGGATCTC ATGAAATCAA AGTTTGGTCG AGCAAGAACA AAGAAAGTGG AAGAGGTGAC GAAGAGAGTA GTCCAAGTTC CGAAGTAGAG ACAGAATTGT TCATTGGGTT GCCTTCTTCT TCAAGAAAGT GA |
| ***BraFT 1*** CDS | ATGTCTTTAA GTAATAGAGA TCCTCTTGTG GTAGGGAGAG TTGTAGGAGA CGTTCTTGAA TGTTTCACAA GATCAATCGA TCTAAGGGTT ACTTATGGCC AAAGAGAGGT GACAAATGGG TTGGATCTAA GGCCTTCTCA AGTTCTCAAC AAGCCAAGAG TTGAGATTGG TGGAGAAGAC CTAAGGAACT TCTATACTTT GGTTATGGTG GATCCAGATG TTCCAAGTCC TAGCAATCCT CACCTCCGAG AATATCTTCA CTGGTTGGTG ACTGATATCC CAGCGACAAC TGGAACAAAC TTTGGCAATG AGATTGTGTC TTACGAGAGT CCAAGGCCCA ACTCGGGTAT TCATCGTATC GTGCTCGTAT TGTTCCGACA GCTCGGTAGG CAAACAGTGT ATGAACCAGG ATGGCGCCAA CAATTCAACA CTCGTGAGTT TGCTTCCCTA TACAATCTCG GCCTTCCCGT GGCTGCGGTT TTCTACAATT GTCAGAGGGA GAGTGGCTGC GGAGGACGAA GAAGTTAG |
| ***BraFT 2*** CDS | ATGTCTGTAA ATAACAGAGA TCCTCTTGTG GTAGGGGGAG TGATAGGGGA CGTTCTTGAA CGGTTCACAA GATCAATCGA TCTAAGGGTT ACATACGGCC AAAGAGAGGT GACAAATGGG TTGGATATAA GGCCTTCTCA AATTATCAAC AAGCCAAGAG TTGAGATTGG TGGAGAAGAC CTAAGGAACT TCTATACTTT GGTTATGGTG GATCCAGATG TTCCAAGTCC TAGCAACCCG CACCTTCGAG AATATCTCCA TTGGTTGGTG ACTGATATCC CTGCGACAAC TGGAACAAAC TTTGGCAATG AGATTGTGTC TTACGAGAGT CCAAGGCCCA CCTCGGGAAT TCATCGTCTC GTGCTGGTAT TGTTCCGGCA GCTCGGGAGG CAAACAGTGT ATGAACCAGG GTGGCGCCCA CAATTTAACA CTCGTGAGTT TGCCGCGCTA TACAATCTCG GCCTTCCCGT GGCTGCGGTT TACTTCAATT GTCAGAGGGA TAATGGCTGC GGAGGACGAA GAACTTAG |
| ***BraFT 3*** CDS | ATGAGACTGA TTTCTCTTAA TGTTACTTAT GGCCAAAGAC AGATAACTAA TGGAATGGAT CTAAGGTCTT CTCAAGTTCT GAACAAACCA ACGGTTGAGA TTGGAGGAGA TGACCTCAGA AATTTCTACA CTTTGGTTAT GGTGGATCCA GATGTGCCAA GCCCAAGCAA CCCGTACCTC CGAGAATACC TCCATTGGTT GGTGACAGAT ATACCTGCCA CAACTGGAAC ATCTTTTGGC AATGAGTTGG TGTACTACGA GAATCCATGT CCCACATCAG GAATTCACCG AGTCGCATTG ATATTGTTCC GACAACTCGG AAGACAAACG GTTTATGCAC CCCAGTGGCG CCAAAGGTTC AACACTCGTG AGTTCGCTGA GAACTACAAT CTTGGTCTCC CCGTGGCTGC TGTTTACTTC AACTGTCAGA GAGAGAATGG CTGCGGAGGA AGAAGGACCT CGTAG |
| ***BraFT 4*** CDS | ATGTCAAGAG AAATAGAACC ACTAATAGTG GGAAGAGTGA TAGGAGATGT CATAGAAATG TTCAATCCAA GTGTGACTAT GAGAGTCACT TACAATTCCA ACACAGTCGT CTCCAATGGT CATGAGCTAG CACCTTCTCT TCTCCTCTCT AGGCCTCGCG TTGAAATTGG GGGCCATGAT CTCCGTTCAT TCTTCACCTT AATCATGATG GACCCTGATG CCCCGAGTCC TAGTAATCCT TACATGCGTG AATATCTGCA TTGGATGGTG ACAGATATCC CCGGGACAAC CGATGCTTCT TTTGGGAGAG AGATGGTGAG ATACGAAACG CCTAAACCTG TGATTGGGAT ACACAGATAC GCGTTTGTGC TGTTCAGACA GAGAGGGAGA CAAACGGTGA AGGCGGCACC GGCAACAAGG GAATGTTTCA ACACAAGAGA CTTCTCTGCT TTCTTTGGTC TTTCTCTACC TGTTGCTGCT GTTTACTTCA ACGCCCAACG TGAAACTGCC CCTCGACGAC GTCCTTCTTA CTAA |
