# Supplementary material for: Hydrogen sulfide promotes flowering in heading Chinese cabbage by S-sulfhydration of BraFLCs
Source: Hortic Res. 2021 Feb 1;8:19. doi: 10.1038/s41438-020-00453-3 (PMC7848000; doi:10.1038/s41438-020-00453-3)
Supplement: Supplementary file 3 — Suppl. Table 2 [file 41438_2020_453_MOESM3_ESM.doc]

**Suppl table 2**  Primers for real-time quantitative PCR

| Description | Genes | Primer Sequence（5ʹ-3ʹ，） |
| --- | --- | --- |
| Bra009055 | *BrFLC* 1-F | CCCATGGGGAGGAAGAAACTTGA |
|  | *BrFLC* 1-R | AGCCGGAGAGAGAGTATAGATT |
| Bra028599 | *BrFLC* 2-F | CCCATGGGAAGAAAGAAACTAGA |
|  | *BrFLC* 2-R | GGAGAAGGTGACTTGTCGGCTAC |
| Bra006051 | *BrFLC* 3-F | CCCATGGGAAGAAAAAAACTAGA |
|  | *BrFLC* 3-R | AGCCAAGGGAGTATTGAGAT |
| Bra022771 | *BrFLC* 4-F | CCCATGGGAAGAAAAAAACTAGA |
|  | *BrFLC* 4-R | GCTGCGAAAAGTAAAACCTA |
| Bra04928 | *BrSOCI* 1-1-F | AAAGGATGAGGTTTCAAGCG |
|  | *BrSOCI* 1-1-R | CTCGTTGTAGTTATGGTAAATGGT |
|  | *BrSOCI* 1-2-F | AATAGATGGAACGAGGAAAG |
|  | *BrSOCI* 1-2-R | AACTAAGAAGCAGGATTGTG |
| Bra04927 | *BrSOCI* 2-1-F | GTGAACCTTCATCAACAA |
|  | *BrSOCI* 2-1-R | TCACGCTATTTCCACTAT |
|  | *BrSOCI* 2-2-F | ATAGTGGAAATAGCGTGAGC |
|  | *BrSOCI* 2-2-R | TGCTGGTTAAGTTCTACCAA |
| Bra00392 | *BrSOCI* 3-1-F | GAAGTATTATACATGCATCA |
|  | *BrSOCI* 3-1-R | GTTAACCATTAACCAGTGTA |
|  | *BrSOCI* 3-2-F | AATAATGACATTTACACTGG |
|  | *BrSOCI* 3-2-R | CCATGTATTTTGATTGATTT |
| Bra00393 | *BrSOCI* 4-F | CACATATTACTATTTGCTCTTC |
|  | *BrSOCI* 4-R | TTCTCGTTGTAGTTATGGAC |
| Bra039324 | *BrSOCI* 5-F | GTGCGAGTCTTGTCTTTTGT |
|  | *BrSOCI* 5-R | GCGACGAGAAGATGATGAAC |
| Bra022475  Bra004117  Bra015710  Bra010052  BraActin | *BrFT1-1-F*  *BrFT1-1-R*  *BrFT2-2-F*  *BrFT2-2-R*  *BrFT3-3-F*  *BrFT3-3-R*  *BrFT3-4-F*  *BrFT3-4-R*  *BrFT4-5-F*  *BrFT4-5-R*  *BrFT4-6-F*  *BrFT4-6-R*  *BraActin-F*  *BraActin-R* | CGAGGAGATCAATAGATGCTCT  ACCATCCATTTGTTCTCACCAG  AAAAATGCCGGGTACTTGACC  ACGAGAGAAGCGAGTTGTAAAAT  CATCTAAGACCTCAAGGCC  GACTTGTAGTTCAAGTTGGAC  ATCTAAGACCTCAAGGCCT  CGATAAGAGGGTCTCTCG  GGATTATGGAATGTTAGATTTCGA  GTGATTCTTCTATCTCTAGAACTT  GTGCTAGAGCATTAAGATAAACACC  ATGGTGTAGTGTGTGTTGTGG  TAGTGTTGTTGGTAGGCCAAGACAT  GGAGCTCGTTGTAGAAAGTGTGATG |
